# Wave–turbulence interaction-induced vertical mixing and its effects in ocean and climate models

**DOI:** 10.1098/rsta.2015.0201

**Published:** 2016-04-13

**Authors:** Fangli Qiao, Yeli Yuan, Jia Deng, Dejun Dai, Zhenya Song

**Affiliations:** 1 First Institute of Oceanography, State Oceanic Administration, Qingdao, 266061 China; 2 Laboratory for Regional Oceanography and Numerical Modeling, Qingdao National Laboratory for Marine Science and Technology, Qingdao, 266071 China; 3 Key Laboratory of Marine Science and Numerical Modeling, State Oceanic Administration, Qingdao 266061, China

**Keywords:** turbulence, ocean surface wave, wave–turbulence interaction, vertical mixing, field measurements on turbulence

## Abstract

Heated from above, the oceans are stably stratified. Therefore, the performance of general ocean circulation models and climate studies through coupled atmosphere–ocean models depends critically on vertical mixing of energy and momentum in the water column. Many of the traditional general circulation models are based on total kinetic energy (TKE), in which the roles of waves are averaged out. Although theoretical calculations suggest that waves could greatly enhance coexisting turbulence, no field measurements on turbulence have ever validated this mechanism directly. To address this problem, a specially designed field experiment has been conducted. The experimental results indicate that the wave–turbulence interaction-induced enhancement of the background turbulence is indeed the predominant mechanism for turbulence generation and enhancement. Based on this understanding, we propose a new parametrization for vertical mixing as an additive part to the traditional TKE approach. This new result reconfirmed the past theoretical model that had been tested and validated in numerical model experiments and field observations. It firmly establishes the critical role of wave–turbulence interaction effects in both general ocean circulation models and atmosphere–ocean coupled models, which could greatly improve the understanding of the sea surface temperature and water column properties distributions, and hence model-based climate forecasting capability.

## 1. Introduction

The ocean surface layer is arguably one of the most complicated dynamic systems. Under the free surface, waves and wave breaking, turbulence and mean shear currents all coexist, acting together and on each other to affect the mixing process downward into the water column below and upward to the air–sea interaction processes above. All those motions are stochastic in nature, on different intertwined scales. Sorting out the detailed dynamics has been a daunting challenge to ocean scientists ever since the beginning of quantitative ocean studies. Their detailed resolution is not in sight even now, and perhaps will not be for the near future. Unfortunately, this layer plays a key role in the coupling of the atmospheric and oceanic systems; we need to find a solution to this problem for the increasingly urgent climate studies.

Of all the motions in the surface layer, it is the surface waves that play a pivotal role. Although they are among the first dynamic phenomena amenable to analytic treatments [1], the details of their generation, evolution and interactions can still not be regarded as completely known [2,3]. Facing such a challenge, the current practice with respect to wave motions is dichotomous: in most studies of large-scale dynamics such as general circulation, on both basin wide as well as regional scales, the surface layer dynamics are glossed over with various parametrization schemes. Wave motions are neglected, being small scale in comparison with the mean circulation systems, as the argument goes. The free surface is regarded as a wall; turbulence dynamics at the surface layer are treated as wall turbulence [4,5]. With careful tuning, the end results seem to be reasonable, but the deficiency becomes obvious when the demand on accuracy becomes high, as in coupled atmosphere–ocean climate modelling [6,7]. On the other hand, wave motions are studied for their role in the detailed air–sea exchanges of mass, momentum and energy, which are the critical boundary conditions for large-scale circulation problems. As the dynamics are too complicated to yield definitive answers, the product of the small-scale studies is also reduced to parametrization. In fact, many of the parametrization schemes of the wave motions were actually based on ocean surface wind rather than on wave parameters [8]. Yet, the cumulative effort of past studies has given us a strong hint that waves could not be neglected in the study of ocean dynamics [6,7,9], not even in large-scale circulation problems, for they are the most energetic motions in the ocean surface layer, consequently playing a dominant role in air–sea interaction processes [10] and in ocean mixing way beyond the surface layer [11].

Even with these understandings, many of the past studies of the waves in the context of ocean dynamics are concentrated on breaking waves [12], for each of the episodic breaking events would release a huge amount of energy. The breaking events are treated as the leading source for turbulence generation at the ocean surface layer that controls the air–sea gas transfer [13]. Further studies, however, reveal that the turbulence conditions so generated are mostly confined to the surface layer with their energy dissipated locally at a depth of the order of the wave amplitude [14]. Their dynamic consequence in the deeper layer is almost negligible. As a result, the non-breaking waves, modelled successfully as irrotational motions, have thus far been treated as totally irrelevant to the large-scale ocean dynamical system, yet studied extensively as an isolated and curious phenomenon in itself. This picture has only recently been challenged by the introduction of non-breaking wave-induced mixing in general ocean circulation modelling through wave–turbulence interactions [6]. Although global circulation modelling results are greatly improved by wave-induced mixing, the derivation of the mixing parameter and the detailed wave–turbulence interaction mechanisms are still not immune from certain contentious questions. The purpose of this paper is to clarify these issues and reconfirm the key role of wave–turbulence interactions in vertical mixing, a critical process for climate and general circulation studies.

The existing literature reports that all the experimental results indicate that the spectra of the turbulence velocity field would have a prominent peak at the frequency of the energy-containing waves, and that the spectral density would be enhanced far beyond the wall turbulence cases over the high-frequency range. Furthermore, all experimental data also indicate an accelerated turbulence dissipation rate when waves are present. The appearance of prominent peaks at the energy-containing wave frequency has been regarded as the prima facie evidence of wave–turbulence interactions that led to the conclusion that the turbulence could not be regarded as independent from wave motions. Yet regarding the consequence of these interactions, we face a high degree of uncertainty. Theoretical studies by Phillips [15,16] and later extended and amplified by Teixeira & Belcher [17] all indicate the important role of turbulence enhanced by surface waves. Additionally, most of the theoretical results are also qualitatively confirmed by detailed laboratory experiments [18]. However, evidence for wave–turbulence interactions from the field remains elusive. For example, a series of observations conducted in Lake Ontario by Kitaigorodskii and Lumley [19], Kitaigorodskii *et al*. [20] and Lumley & Terray [21] led Lumley & Terray [21] to conclude that ‘the shape of (the longitudinal) turbulence velocity spectra (but not the magnitude) in the ocean surface layer could be understood through kinematics without recourse to the dynamics. The phenomenon is said to be the consequence of frozen homogeneous isotropic turbulence convected by the wave orbital velocity’. Granted that the wave energy in the field is many orders of magnitude higher than the turbulence, direct observations of turbulence in the field are extremely difficult. But the critical question of whether turbulence and wave interactions in the field can be accounted for solely through kinematics or through more complicated dynamics still begs a satisfactory answer.

Our open ocean field experiment was designed to answer this challenging question. Furthermore, we also intend to clarify the role of turbulence interactions with non-breaking waves in vertical mixing, and propose a simple parameter to account for their effects for even large-scale geophysical problems as in the general circulation and coupled climate models. Taking advantage of the recently developed Holo–Hilbert spectral analysis (HHSA) [22], we have made the first direct measurement of turbulence in the open ocean. Our results also confirm the mechanism of turbulence enhancement through straining of the vorticity lines of the rotational turbulence motions as originally proposed by Phillips [15,16] and later extended and amplified by Teixeira & Belcher [17]. Further support comes from agreement with the most precisely conducted laboratory experiments by Thais & Magnaudet [18]. The physics of the interactions is exactly as Teixeira & Belcher [17] have pointed out: the net effects of wave-induced straining of the turbulence are through the Stokes drift associated with the wave motions to bend the vorticity lines streamwise and thus enhance mixing. This physical picture is directly confirmed in our field experiments. Additionally, we have proposed a simple and physical way to parametrize the effects of wave-induced mixing. The end product matches almost exactly with that obtained by Qiao *et al*. [6]. The rest of this paper is divided into the following sections: §2 summarizes the mathematics of kinematical and dynamical consequences of wave–turbulence interactions in terms of spectral representations; §3 covers a review of the past experimental results to clarify the physics of wave–turbulence interaction; §4 describes the field experiment and the observational results; §5 presents the newly proposed parametrization scheme and finally §6 is our conclusions.

## 2. The spectral representations of additive and multiplicative interactions

In order to understand the detailed physics of wave–turbulence interactions, it is important to clarify the spectral representations of different interacting mechanisms. For this purpose, it suffices for us to examine the mechanism qualitatively as shown by Huang *et al*. [22], in which they pointed out that the traditional Fourier analysis is incapable of representing the nonlinear multiplicative processes: the problem cannot be circumvented by simply forcing all multiplicative processes into additive ones. This linear representation is the common drawback found in all of the existing mathematical expansions. The critical consequence to the study of dynamics is profound. Fourier representation is routinely used to represent wave–turbulence interaction, for example. However, the dynamics is much more complicated than the simple additive formula used in the Fourier spectral representation. Let us start with the control equations
2.1
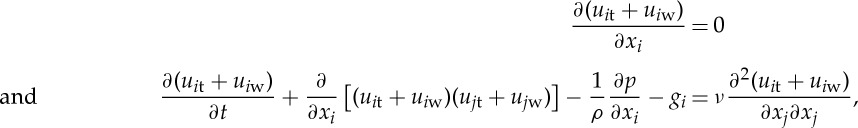
then we will have the multiplicative terms in the momentum equation as
2.2
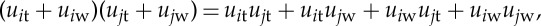
where the subscript ‘t’ stands for turbulence and ‘w’ for wave contribution. On the right-hand side of equation (2.2), the last term represents the dominant wave contribution, whereas the middle two terms represent modulation of the turbulence by the wave motions and the cross-scale interactions. The first term could be neglected in comparison with the others. This is why the spectrum would have a prominent peak at the wave frequency. Of critical importance is that their relationships are all multiplicative.

Indeed, modulation patterns similar to this have been observed by Thais & Magnaudet [23] and will be discussed later. The point we want to accentuate here concerns the spectral representation: the modulation pattern should have no trace of any prominent peak associated with the waves, if the process is purely multiplicative. The multiplicative effect actually spreads the energy of the waves almost uniformly across the turbulence frequency range as explained by Huang *et al*. [22]. The actual processes are much more complicated with a mixture of cross-scale interactions among waves along with turbulence as well as waves with other waves. Such complications are all obscured and concealed by the Fourier analysis. This is one of the main reasons why the details of the wave–turbulence interactions have been elusive. Before getting into the new approach, let us start with a review of past experimental studies.

## 3. A review of past experiments on wave–turbulence interaction physics

There have been many attempts to measure wave–turbulence interactions directly. To review the progress logically, we decided to divide the discussions by subject area rather than chronologically as follows: (i) the kinematics: methods to separate turbulence and waves, (ii) the dynamics: the physics of wave–turbulence interactions, and (iii) the theoretical studies.

### (a) The kinematics

Separating the wave motions from the turbulence is a prerequisite for studying their interactions in detail. Therefore, this topic has been studied by many investigators. The methods cover many approaches, from simple phase averages to more complicated triple-nonlinear separations. Unfortunately, all the different results are not free from some lingering mixture of waves and turbulence. The best that we can do with these older methods remains approximate.

The first attempt to separate the waves from turbulence is by phase averaging [18,24]; the idea is built on the assumption of a periodic motion of the waves. The method is very powerful if the wave is strictly periodic. The phase average is defined as
3.1
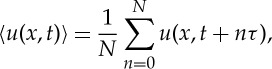
for any quantity, *u*(*x*,*t*), with **τ** as the period of the wave. Indeed, if wave velocity is strictly periodic, then
3.2
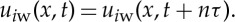
Therefore, any product term involving a wave parameter, the velocity, say, would become
3.3
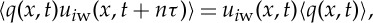
by virtue of (3.1) and (3.2). Unfortunately, strictly steady and periodic waves do not exist owing to inherent dynamic instability [25,26]. Indeed, using phase averaging is a daunting challenge in any real case. Let us use the mechanically generated wave in the laboratory as an example. The data used by Huang *et al*. [2,27,28] are generated by a mechanical wave-maker, measured at different fetches from it. The instability of a Stokes wave would make the phase impossible to follow. Indeed, the phase average is eschewed by most investigators. The only exception was the study by Thais & Magnaudet [18], who succeeded because they had employed mechanically generated waves under a light wind, which is actually a stability combination. Because this is one of the most important studies of wave–turbulence interactions available, we will return to it shortly.

Kitaigorodskii & Lumley [19] had attempted it but failed; they had to switch to linear filtering. Linear filtering or fitting was proposed by Donelan *et al*. [29], in which the linearized kinematical and dynamical boundary conditions are used to derive a velocity potential, *Φ*(*x*,*t*), to fit the measured surface elevation data, *ζ*(*x*,*t*)
3.4
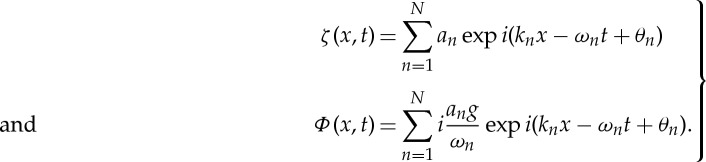
Here, the assumption of irrotational waves has been made. The linear fitting is only an approximation, for all the nonlinear effects of the waves are designated as due to turbulent motion; therefore, the separation is incomplete.

To remedy this shortcoming, Thais & Magnaudet [23] proposed the nonlinear triple decomposition method (TDM). Here, they even removed the assumption of irrotational wave motion, but they do invoke the assumption of no correlation between wave elevation and the turbulence velocity field. The method assumes that the total velocity could be decomposed into three parts:
3.5
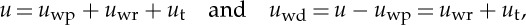
where the subscripts ‘wp’, ‘wr’, ‘t’ and ‘wd’ stand for potential wave, rotational wave, turbulence and wave difference, respectively. They used the full nonlinear equation to compute a velocity field best fitting the surface elevation data. Then, they computed the spectrum of the rotational wave contribution by
3.6
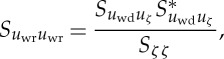
and finally obtained
3.7
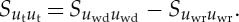
In arriving at this result, they have invoked the following assumptions:
3.8
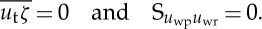
The first assumption seems to be reasonable, for the weak turbulence would not have any surface signature anyway, but the second assumption might be somewhat problematic. If the elevation consists of the sum of rotational and irrotational waves, how can we be sure that they are uncorrelated spectrally? Furthermore, how can a wave be both rotational and irrotational? Although a recent experimental study by Monismith *et al*. [30] has provided some circumstantial evidence to show that waves are indeed rotational, based on the drift current characteristics associated with the waves, a detailed dynamic frame of the rotational wave is still lacking. Therefore, it is fair to conclude that presently we do not have a satisfactory method to separate the turbulence from the coexisting random wave motions. Fortunately, we do not have to take a stand on this issue, for their experimental data on the turbulence properties under mechanically generated waves under light wind had provided a rich cache of data to help us appreciate the wave–turbulence interactions. Let us now switch to the dynamics studies.

### (b) The dynamics

Two sets of experimental studies are of critical importance to our understanding of the dynamics of wave–turbulence interactions. The first set was a field study in Lake Ontario by Kitaigorodskii & Lumley [19], Kitaigorodskii *et al*. [20] and Lumley & Terray [21]. The turbulence was measured from the surface down to a 6 m depth by a strain-sphere mounted on a tower 1.6 km from the coast. Turbulence statistics are extracted from the velocity data using linear filtering. As the separation is incomplete, all turbulence spectra retain a prominent peak at the energy-containing wave frequency. This general form led Lumley & Terray [21] to conclude that turbulence velocity spectra in the ocean surface layer could be understood without recourse to the dynamics. The kinematics, however, could not explain the more important fact that the turbulent velocity spectra measured beneath the wind waves show a large enhancement about the central wave frequency with an apparent increase in spectral density at high frequencies. This not too subtle enhancement of the spectral density is the true consequence of dynamic interactions, as in the wave-induced modulations discussed by Huang *et al*. [22]. Limited by the separation method employed here, their conclusions for the study, though of critical importance, could only be regarded as questionable. The true physics of the dynamic turbulence and wave interactions could be understood more quantitatively through the precisely controlled laboratory study by Thais & Magnaudet [18].

Thais & Magnaudet [18] reported their controlled laboratory experiments conducted in the large Air–Sea Interactions Facility of the ‘Institut de Mécanique Statistique de la Turbulence’ (now IRPHE-IOA) in Marseille. The dimensions of the test channel are 40 m long, 2.6 m high and 3.2 m wide. Both wind waves and mechanical waves were used in the experiments. The part most important to our understanding of wave–turbulence interactions are the measurements made at 26 m fetch with mechanically generated waves at 1.0 Hz and an amplitude *a*=27.0 mm (wave slope *ak*=0.106). The velocity components were measured with a two-dimensional laser Doppler velocimeter (LDV) to obtain dimensional velocity data to be within 2–4 mm from the wave-troughs. Wave elevations were independently measured near the LDV probe. Light winds (3.0, 4.5 and 5.8 m s^−1^) were also used. This range of wind speeds actually could help in stabilizing the mechanically generated wave from the amplitude modulations (AMs) [31]. The wave–turbulence velocity was separated by the nonlinear TDM [23] as presented above. The most salient results they reported are these: first, the wave motion in the ocean is too big for us to measure the turbulence directly, so that the laboratory setting offers some advantage here. Second, wave–turbulence interactions greatly affect energy transfers over a significant frequency range, besides enhancing the turbulent kinetic energy greatly as well. Finally, the turbulent statistics and their modulation with respect to the wave phase show that turbulence can be modulated by the wave-induced motions over a wide range of scales, resulting in turbulence production at the wavy region above the troughs. The scale of waves generated in the laboratory is necessarily small, making the scale separation difficult. It is to their credit that their carefully planned and executed experiments succeeded in extracting such valuable results under so difficult conditions.

Given the meticulous care they have exercised over the experiments and data analysis, it is tempting to conclude that these sets of experiments might represent the ultimate level that one could achieve in the laboratory study of wave–turbulence interactions. The basic physics seems to be clear now. Yet the inability to separate the turbulence from the wave motions in the field still begs additional questions. Could the wave–turbulence interactions be so strong that the dynamic consequences are beyond the simple additive and multiplicative interactions discussed by Huang *et al*. [22]? Let us turn to the theoretical study.

### (c) The theoretical study

Thus far, the most detailed theoretical study on wave–turbulence interactions was conducted by Teixeira & Belcher [17]. They have employed a moving Lagrangian frame under the assumptions that the orbital velocity is much larger than the turbulence intensity, and the straining of the turbulence by the wave is much larger than the straining of the turbulence by itself. Their detailed analysis led to the following conclusions: first, the vorticity in the turbulence is modulated by the wave orbital motions directly. The dominant effects are due to the Stokes drift over several wave periods indirectly, and that the Stokes drift would produce a shear stress working against the wave orbital motions, causing the wave decay. Recently, Benilov [32] discussed the possibilities of generating turbulence by the vortex instability of the potential surface waves. The small initial vortex perturbations of potential surface waves always grew and the interaction between the vortex perturbations and the potential waves produced small-scale turbulence.

It should be pointed out that although they claimed that the effect of the orbital motions having a straining rate of *O*(*akω*) was *direct and of* first order, it is relatively weak, because the wave motions are periodic and the total strain could never exceed *O*(*akω*). But the indirect effect of the Stokes drift, whose straining rate can be estimated as *O*(*a*^2^*k*^2^*ω*), is of second order. It is cumulative, and the total strain is of *O*(*a*^2^*k*^2^*ω*t), which would grow with time *t*. This argument should be amended for the direct effect, which could also be cumulative, because the wave orbital velocity is never a closed loop in a random wave field. The open-end straining effect would not be exactly reversible; thus, its consequences could at least be equal to, if not stronger than, the indirect effect proposed here.

The cumulative results of all the past studies suggest to us that the wave–turbulence interaction should be a dominant mechanism, but direct field observations are still elusive. Part of the difficulty is due to not using the proper analysis method on the data. Taking advantage of the recently developed HHSA, we have embarked on a field test to see if we can indeed directly observe the wave–turbulence interactions under field conditions.

## 4. The field experiments and results

**Figure 1.**
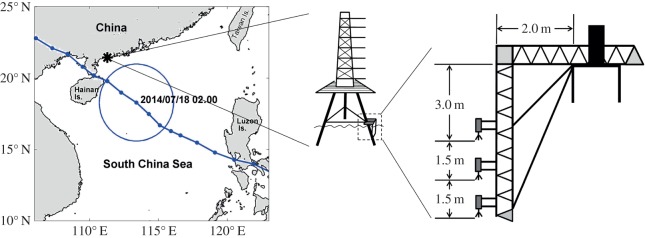
The experiment site and the instrument installation locations. Typhoon Rammasun passing through the area during our experiment, whose location on 18 July 2014 is marked by the circle, when instruments were damaged.

The experiment was conducted at the Observation Platform of Marine Meteorology (OPMM) tower located at 21°27′ N, 111°18′ E, at Bohe, Guangdong, China (figure 1). The tower is in water of 16 m depth and 7 km from the nearest shore. The experiment was conducted over the period from 13 to 21 July 2014. The following measurements were made: three acoustic Doppler velocimeter (ADVs, Nortek Vector) were attached to the tower at vertical intervals of 1.5 m, with the upper ADV at 2.6 m below the mean water level. In order to avoid the side effects of supporting structure on the observed data, a hollow triangle column was designed to support three ADVs. The hollow structure could let the water flow fluently with less perturbation. The side length of triangle was 20 cm, and the diameter of the steel member was 2.0 cm. The ADVs were arranged 30 cm away from the triangle structure. To cover the full experiment duration with limited memory, measurements were made every 30 min for 3 min duration at the sampling rate of 64 Hz for the upper and lower ADVs, and 8 Hz for the middle one. The data were stored in a recorder encased in the instrument. Should any changes be made to the experiment, the recording cables were also connected with all the three ADVs, so we could save the observed data to computer as we wanted. Other ancillary measurements are discussed in the electronic supplementary material. Soon after the instruments were installed, Rammasun, a category 4 typhoon passed by and made landfall over the nearby Hainan Island on 18 July 2014. Packing a 10 min sustained wind speed of 165 km h^−1^ (1 min sustained wind at 250 km h^−1^), the typhoon proved to be extremely destructive. Although the OPMM tower survived intact, the experimental settings were totally demolished. All instruments were damaged beyond repair or lost when this typhoon passed through the area. Fortunately, data from the lower ADV instrument could be salvaged from the recovered recorder covering about 185 h, from 17.00 13 July to 10.00 21 July, but only the data covering about 108 h, from 17.00 on 13 July to 03.30 on 18 July, can be used for further analysis, because the heading, pitch and roll of the lower ADV had shown that the frame-attached ADVs with OPMM began to waggle owing to the effect of Rammasun since 03.30 on 18 July. These are the data used in this paper; more detailed experimental and meteorological conditions can be found in the electronic supplementary material.

Two typical sections of data are presented here. One section was recorded at 15.00, 15 July 2015, well ahead of the typhoon, and is called Data section 1 in the following. The other section was recorded at 17.30 on 17 July, just before the instrument was damaged. We call it Data section 2. The crucial wave–turbulence interaction mechanism is reported here.

**Figure 2.**
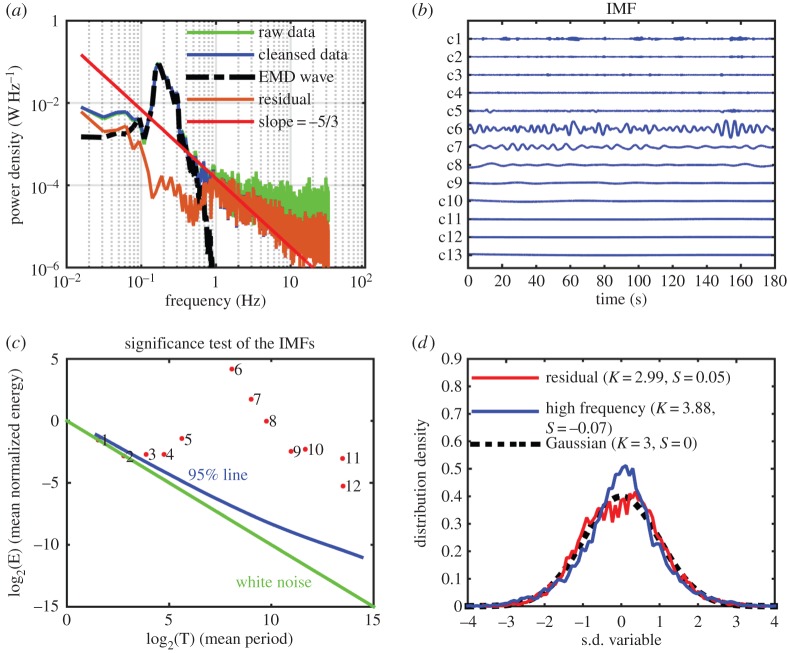
(*a*) Various Fourier spectra, the raw and cleansed data, the part for waves and the residue after the wave motions have been removed through EMD, of the Data section 1 collected at 15.00, 15 July 2014, way ahead of the typhoon. (*b*) The IMFs of the cleansed data: the wave motions can be seen in components 6 and 7. (*c*) The significance test of Data section 1: components 1 and 2 have the same characteristics of white noise; components 3, 4 and 5 are beyond the white noise and should contain information; components 6 and 7 are the most energetic wave motions; and the rest are for the large-scale motions. (*d*) The probability distribution density of various data components: it is intriguing that the residue (thin solid line) containing all motions except waves behaves near-Gaussian, whereas the high-frequency turbulence and noise (thick solid line) only is non-Gaussian. The Gaussian model is given by the dotted line.

For Data section 1, we will concentrate on the vertical component of the velocity measurements, for by construction the set-up Doppler instrument performs best along this direction. The data from the horizontal components are presented in the electronic supplementary material, for our analysis indicates that these data are only marginally useful for our purposes. The detailed data analysis procedures are also given in the electronic supplementary material; we will outline the data analysis of the vertical components here as follows: the raw data from the vertical component was first cleansed by removing the occasional outliers caused mostly by data dropout for lack of suspended particles serving as acoustic reflectors. The root mean squared value of the cleansed data is 0.10 m s^−1^, which suggests that the magnitude of the velocity is about O(10^−1^ m s^−1^). The Fourier spectra for the raw and cleansed data are given in figure 2*a* together with a Kolmogorov −5/3 power law spectral form used as a guide for turbulence motions. From the spectra, we can see that, after the removal of the outliers, the spectrum of the cleansed data generally follows the Kolmogorov spectral line up to near 10 Hz before finally levelling off. Ideally, the higher the frequency, the more isotropic the turbulence should be. The levelling off, however, indicated otherwise; the higher-frequency turbulence seen here actually approaches white noise, a fact that can only be explained by the insufficient accuracy of the ADV instrument used in this experiment. The overall spectral shape is similar to those obtained either in the laboratory [18] or in the field [20], all showing a prominent peak of the surface wave, near 0.2 Hz in this case, and a long tail. Of note here is that the spectral tail, covering nearly two decades in frequency, indicates that the large-scale waves in the open ocean data indeed offer, for the first time, a possibility to have a scale separation between waves and turbulence.

**Figure 3.**
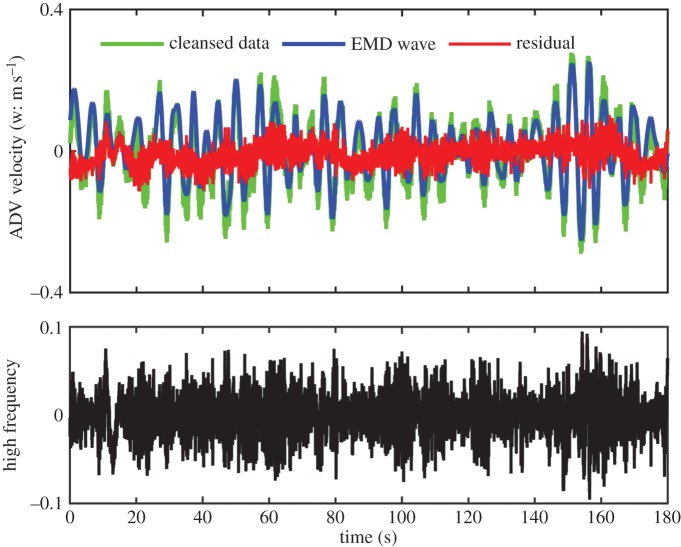
The data and the separation of waves and turbulence components: the cleansed data (light green) and the wave motions (blue) reconstituted by components 6, 7 and the final trend to give it the right level; the residue (red) defined as the difference between the data and the wave motions; and, finally, the high-frequency turbulence and noise given in the lower panel.

To separate wave motion from turbulence, we used the empirical mode decomposition (EMD) method as a filter [28], which can decompose the data into a series of intrinsic mode functions (IMFs) and a residual trend varying monotonically. Specifically, given a time series *x*(*t*), EMD can decomposes it into the form of 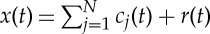 through the sifting process, where *c*_*j*_(*t*) is the *j*th IMF and *r*(*t*) is the trend. Generally, *c*_*j*_(*t*) is physically meaningful and can be written as 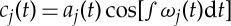 with *a*_*j*_(*t*) and *ω*_*j*_(*t*) representing the amplitude- and frequency-modulated functions of time, respectively. EMD is an adaptive and temporal local analysis method that is especially effective for non-stationary and nonlinear problems. In this paper, the cleansed data are decomposed by EMD into 12 IMFs shown in figure 2*b*. Wave components are clearly visible in components 6 through 7. A significance test [33] is then conducted, and the results are given in figure 2*c*. From the significance test, we can see that components 1 and 2 should be treated as noise, for they have the same characteristics of pure white noise. These components also fall into the levelled off region of the spectrum. IMF components 3 and higher should contain information. The most prominent components in terms of energy density here are components numbered 6 and 7 covering the frequency of the spectral peak range. Those components are designated as waves. The sum of the IMF components from 6 to 7 and the trend is used as wave motions, shown as the blue line in figure 3. The residue (shown in red) after the removal of the waves should be noise, high-frequency turbulence and large-scale ocean motions that include local wind shear, mean and tidal currents, and even some large-scale turbulence and waves other than those associated with the spectral peak. From this residue, the components with frequency higher than the waves are extracted, and shown in a separate panel of figure 3. These components are the primary candidates for our investigation of the wave–turbulence interactions directly. This wave–turbulence separation is based only on EMD without explicit dynamical considerations. As EMD can separate components with physical significance, we believe the dynamics are in fact the determining factor implicitly. The results of this separation are also shown in the spectral from in figure 2*a*. Granted that the wave–turbulence interactions are nonlinear in nature, this additive extraction should not be expected to be complete. This is why the removal of the wave components shows a slight overestimation that causes a dip in energy of the residue spectral line. As the wave motion is many orders of magnitude higher than the turbulence, a very good approximation is to use the additive method near the peak. This separation seems to be effective and quite clean.

We then conducted a probability test on the separated components, and found some curious results, as given in figure 2*d*. The sum of the higher-frequency components (IMF no. 1–5), presumably the turbulence components, shows a highly non-Gaussian characteristics with skewness at −0.07 and kurtosis at 3.87. On the other hand, the full residue consisting of all motions other than the surface waves has a near-Gaussian distribution with skewness at 0.05, and kurtosis at 2.99 instead of 3.00. The one explanation we can advance for these results is as follows: the higher-frequency components are indeed turbulence, but they are constantly under the nonlinear cross-scale straining and modulation by the surface waves. Therefore, their behaviour is highly nonlinear and hence their distribution is by itself highly non-Gaussian. If we take the overall turbulence field including all the scales, with some even larger than the surface waves, the distribution is Gaussian as expected. Another possibility is the shallowness of the water at only 16 m. For the surface waves of 50–100 m in length (peak frequencies 0.2–0.1 Hz), the waves are indeed in the shallow water range. In addition, the presence of the free surface altered the structure of turbulence drastically, making it strongly anisotropic in the whole water column. These details are worthy of our attention in the future. Longer data might make the distribution smoother, but we believe that the basic properties and conclusions would not be changed. Next, we will examine the details of the wave-caused straining and modulations.

**Figure 4.**
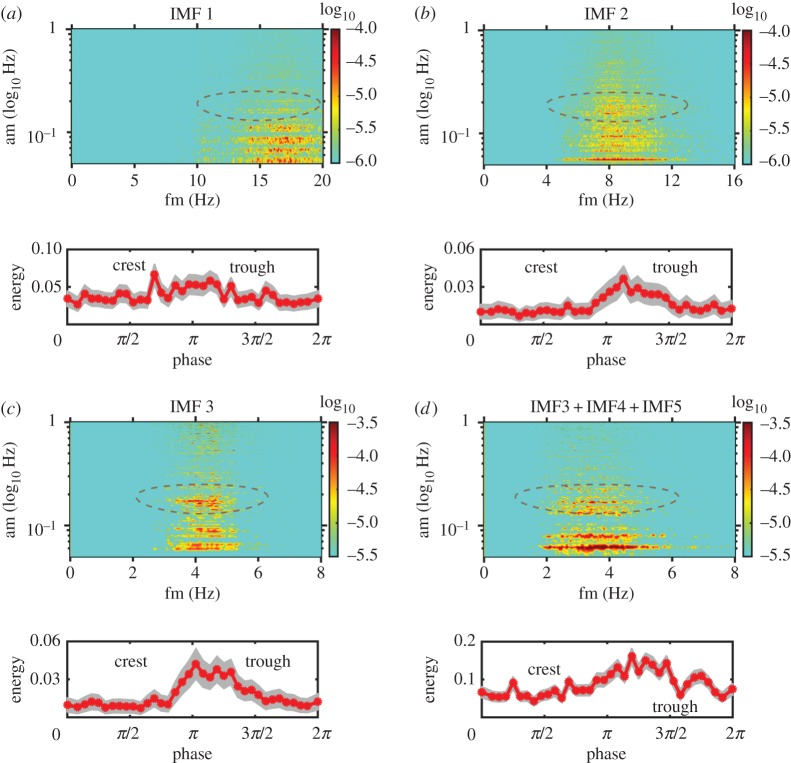
The Holo–Hilbert spectral analysis and phase locking test of Data section 1. (*a*) The Holo–Hilbert spectrum of IMF 1 (upper panel) and the phase distribution (lower panel) with the grey zone indicating±s.d. confidence bound: note that this component should be white noise based on the Fourier spectral analysis, then the significance test. Now, the phase distribution is also uniform, which also indicates that this component is indeed noise. (*b*) The same as in part (*a*), though this IMF is still in the noise range, it is moving towards the true signal in the Fourier spectrum. Here, the energy density in the Holo–spectrum starts to show modulation by the wave motion (circled with ellipse around AM near 0.2 Hz), and the phase distribution starts to show the concentration toward to the trough. (*c*) The same as in part (*a*); this is the first IMF components containing genuine information based on Fourier spectrum, significance test and now the Holo–Hilbert spectrum also indicates strong modulation and phase distribution concentrated near the trough region. (*d*) The sum of IMF 3, 4 and 5. The Holo–Hilbert spectrum showed clearly the wave modulation of the high-frequency turbulence, and the phase distribution is also concentrated in the transfer region from crest to trough.

We have the data and the tools to study the wave straining and modulation on turbulence directly using the HHSA for each IMF components with the results shown in figure 4*a*–*d*, together with the phase distribution of the turbulence components. Figure 4*a* is for the first IMF with a frequency range from 15 to 20 Hz. Both the significance test and the Fourier spectrum indicate that this component should be like white noise. Indeed, the phase distribution, given in the figure of the energy variation with respect to the phase of the surface waves and shaded with 1 standard deviation error bar, is nearly uniform. Thus, all three tests corroborate to identify genuine turbulence and white noise from the instruments. Figure 4*b* is for the second IMF with a frequency range of 7–10 Hz, which is almost in the Kolmogorov spectral range from the Fourier spectrum. Here, we can see that the AM by the dominant wave is starting to be visible, but the significance test disqualifies it as bona fide turbulence motions. When we proceed to the third IMF shown in figure 4*c* with frequency between 3 and 5 Hz, we can see a clear wave AM effect around the spectral peak frequency. The significance test also indicates that this component should contain information above and beyond the white noise level. The phase distribution shows a clear enhancement of energy density from crest to trough, which is similar to the laboratory results by Thais & Magnaudet [18]. It should be pointed out that the significance of the AM modulation by frequency lower than the surface wave is still unknown; it might be caused by the wave groups as suggested by the IMF variations in figure 2*b*, but the exact causes should be explored in the future. The above-mentioned analyses illustrate that what we have shown here is consistent with the theoretical predictions and the laboratory observations of wave–turbulence interactions. The same pattern persists to IMF components 4 and 5, which are not shown in the paper. All the indications from the significance test, the Fourier spectrum and the phase distribution concur to show that these components are indeed modulated by the dominant surface wave at its frequency. If we sum all the relevant IMF components together, i.e. IMF3–5, then we would get the result shown in figure 4*d*. This sum covers the strong AM modulation for the turbulence frequency range over one decade, from nearly 2 to 5 Hz and a phase distribution showing an enhancement from crest to trough. Curiously, though the shallowness of the water made the turbulence structure anisotropic, spectral shape in this range still agrees with the Kolmogorov, a fact worthy of further investigations.

**Figure 5.**
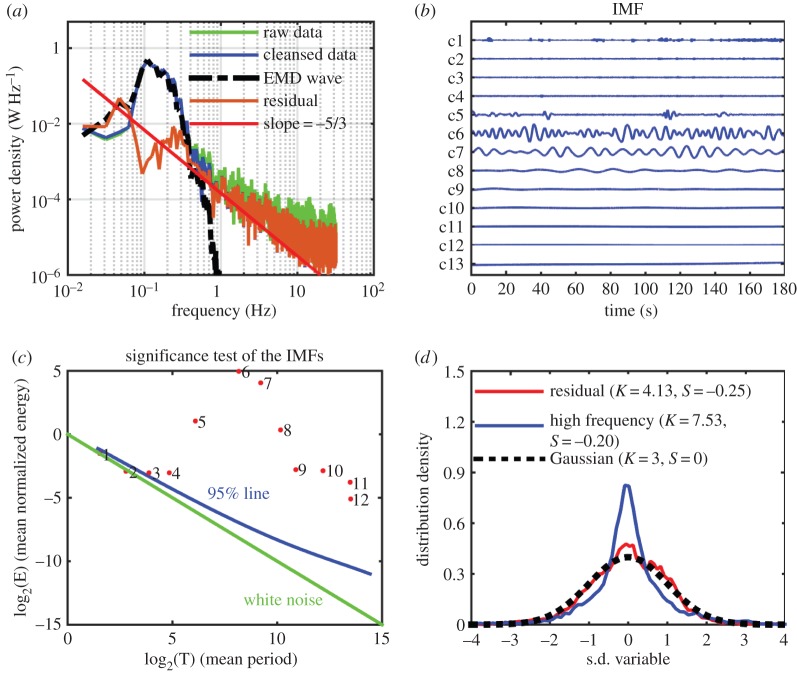
(*a*) The same as figure 2*a*; here the data are from Data section 2, when the experiment site was under the influence of typhoon Rammasun. Note the broadening and down shift of the wave energy in the Fourier spectrum. (*b*) The same as in figure 2*b*; note the wave motions have now spread to IMF components 5–7. (*c*) The same as in figure 2*c*; here the significance also indicates that the components 5–7 are energetic and information containing. (*d*) The same as in figure 2*d*; the highly nonlinear typhoon-induced waves have distorted all motions in the ocean. As a result, the probabilities are highly non-Gaussian now.

**Figure 6.**
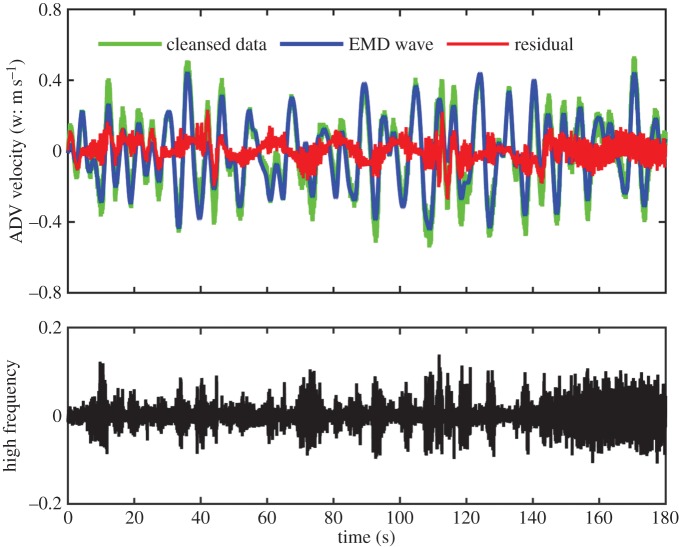
The same as in figure 3; here the broadening of the wave motion part of the spectrum has caused some leakage into the high-frequency turbulence part of the data.

**Figure 7.**
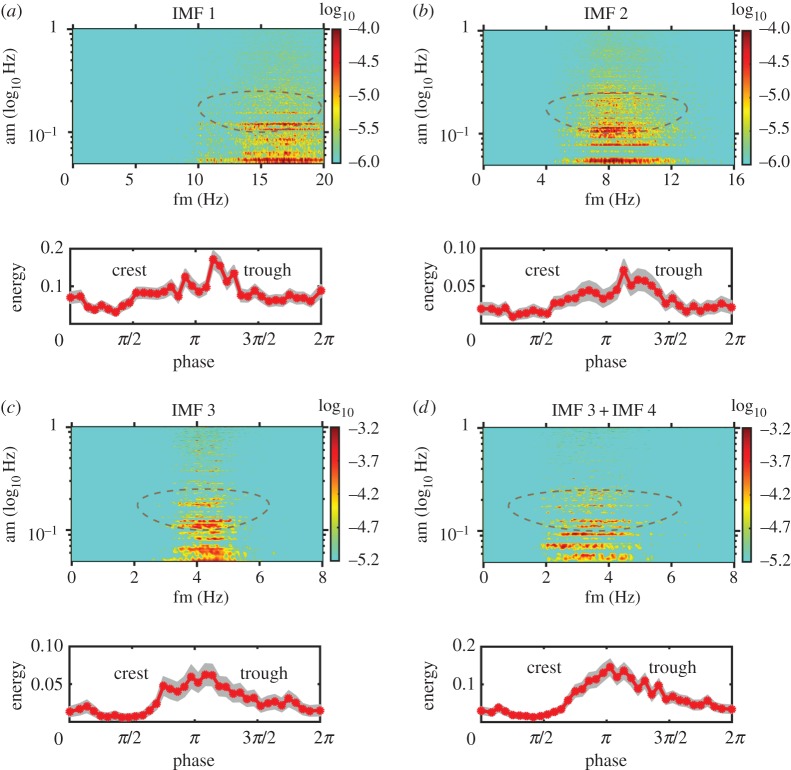
The Holo–Hilbert spectral analysis and phase locking test of Data section 2. (*a*) The same as in figure 4*a*; note that broadening of the wave motion part of the spectrum makes the high-frequency motion almost into the turbulence region. Now the phase distribution also shows some trough concentration. (*b*) The same as in figure 4*b*; the effects of the broadening of the wave motion part of the spectrum is even clearer. One can classify this as high-frequency turbulence, except that it failed the significance test. (*c*) The same as in figure 4*c*; this is the first IMF component containing genuine information based on the Fourier spectrum, significance test and now the Holo–Hilbert spectrum also indicates strong modulation and phase distribution concentrated near the trough region. (*d*) The sum of IMF 3 and 4. The Holo–Hilbert spectrum showed clearly the wave modulation of the high-frequency turbulence, and the phase distribution is also concentrated in the transfer region from crest to trough.

Next, we consider Data section 2, which was collected when the effect of typhoon Rammasun was near, and just before the instruments were damaged by the strong forces. The sea state has increased drastically. The root mean squared value of the cleansed data in this case is 0.21 m s^−1^. The corresponding Fourier spectrum, IMF components, significance test, wave–turbulence separation and the probability results are given in figures 5*a*–*d* and 6. From figures 5 and 6, we can see that the surface wave spectrum has broadened considerably to near, or even slightly lower than, 0.1 Hz. The IMF and significance test indicate that the energy density increased from IMF component number 5, which also contains some visible wave effects from the IMF data. Therefore, it would be treated as waves and large-scale motions. Again, the first two IMFs are no different from white noise, leaving only IMF components 3 and 4 as candidates for high-frequency turbulence modulated by and interacting with the surface waves. For the probability density, the full residue already deviates from a Gaussian distribution with skewness at 0.25, and kurtosis at 4.13 instead of 3.00. The sum of the high-frequency turbulence components has skewness at −0.20, and kurtosis at 7.53. Longer data would certainly make the distribution smoother, but we believe that the effects of the highly nonlinear typhoon-whipped waves are the main cause for these nonlinear characteristics. The HHSA results are given in figure 7*a*–*d*. The similar phase-locked AM modulation of the turbulence is again clearly revealed.

The sum of these results reveals, for the first time, that direct modulation of turbulence by surface waves under field conditions is possible, if proper data analysis methods are employed. This is also the first time that wave–turbulence processes have been observed directly in a field experiment. All the results are in agreement with the theoretical results by Phillips [15,16] and later extended and amplified by Teixeira & Belcher [17], as well as the carefully conducted laboratory experiments by Thais & Magnaudet [18]. The wide separation of the wave and turbulence scale enables us to see the detailed straining and modulation directly.

## 5. A proposed parametrization for vertical mixing and its applications to ocean and climate models

Based on our review of past experimental and theoretical studies and the present field experiments, the physics of wave contribution to mixing is clear: there is strong interaction between turbulence and waves. The physical mechanism is directly through the wave orbital velocity modulations, and indirectly through the Stokes drift coupled with the large excursion of the wave-induced particle motions, and the later indirect Stokes drift effect is much more important. Based on this physical picture, we can propose a phenomenological viscosity/diffusivity based on dimensional considerations as
5.1
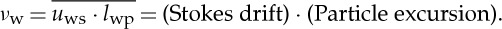
The Stokes drift for a random wave field has been derived by Huang [34] as
5.2
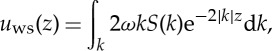
whereas the particle excursion is simply
5.3
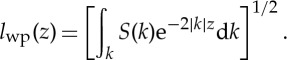
Therefore, by (5.1), the non-breaking surface wave-induced vertical viscosity should be
5.4
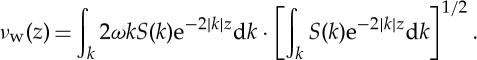
Amazingly, this expression is of exactly the same form as the one proposed by Yuan *et al*. [35] and Qiao *et al.* [6]. In their original derivation, they have also invoked mixing length theory and wrote
5.5
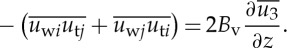
The non-breaking surface wave-induced vertical viscosity was expressed as
5.6
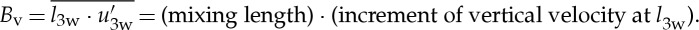
They had selected the particle excursion as the mixing length, same as in (5.4). For the velocity, they selected
5.7
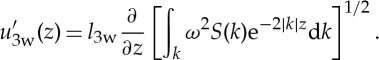
The end product is
5.8
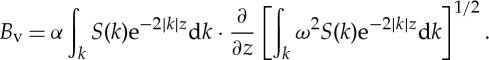
In their approach, the importance in the physics of mixing is distracted by the formality of the existence of the wave–turbulence Reynolds stress given on the left-hand side of equation (5.5), which has aroused considerable controversy. Given that our ability to quantify the wave–turbulence interactions is better now, though still incomplete, the existence of the non-zero correlation between turbulence and waves cannot be resolved at this time. We believe it is more likely for the correlation to be non-zero for the following reasons: first, the existence of the strong wave–turbulence interactions is beyond doubt, as indicated by the enhancement of the high-frequency velocity spectra both in the laboratory and in the field. Therefore, the waves could indeed impart some wave characteristics in the turbulence velocity field, albeit a second-order effect. The existence of spectral energy density does not immediately imply a non-zero mean. In our present case, however, we have identified that physics of the mixing is due to the non-zero mean Stokes drift. However, second-order effects on waves might be as important as turbulence effect. Second, the waves might not be totally irrotational owing to the finite but non-zero viscosity of water. Indeed, the most recent study by Monismith *et al*. [30] has provided circumstantial evidence to support such a claim. The final resolution is still to be achieved.

There might be a slight difficulty in their previous derivation: the starting point of (5.5) clearly indicates that the non-breaking surface wave-induced vertical viscosity is the consequence of the ‘wave–turbulence Reynolds stress’; therefore, the final expression for the non-breaking surface wave-induced vertical viscosity should involve both turbulence and wave-related parameters. Yet, it is given only in terms of wave parameters. In the present approach, we have only argued on the wave-induced effects. In a region of the ocean where wave energy is dominant, we argue that the process should be expressed in terms of waves only. Field observations consistently show that the wave energy is more than one order of magnitude larger than turbulence, which makes the measurement of turbulent statistical properties impossible, especially under the handicap of improper analysis tools. Controlled laboratory experiments also show quantitatively that the wave effects are indeed an order of magnitude larger than the ‘wall turbulence’ analogy. Here, we have shown the evidence of wave–turbulence interactions directly in the field. In the open ocean, no other motion can compete with the surface wave in terms of energy content [11] in the surface layer down to the depth of the order of the wavelength of the energy-containing waves (from tens of metres to a few hundred metres). As the wave effects decay exponentially, beyond that depth, shear current and internal waves might become important. As vertical mixing at the surface layer is an energy-related problem, we believe the wave-dominant mixing parameter is physically and logically sound.

Extensive tests of this mixing parametrical scheme [6,7,36–38], including intercomparisons with well-established mixing parametrical schemes such as KPP [5] and the turbulence closure approach [4], indicate that the addition of the present mixing parametrical scheme is clearly better than the other approaches. Considering that the overwhelming energy flux across the air–sea interface is going into waves, and that the mixing is an energy-dominating problem, we believe that for any parametrical scheme to make physical sense, it should include wave parameters explicitly. Physically, the processes is simple and clear: the wave-induced Stokes drift causes a shear, which is a more prevailing motion in the ocean surface layer than the turbulence-induced shear based on controlled laboratory experiments [18]. Indeed, this wave-induced drift is also a driving mechanism for Langmuir circulation [34,39–41], which is also a crucial factor in surface mixing. Therefore, it makes eminently physical sense for a parametrization of the non-breaking surface wave-induced vertical viscosity to include the Stokes drift explicitly.

**Figure 8.**
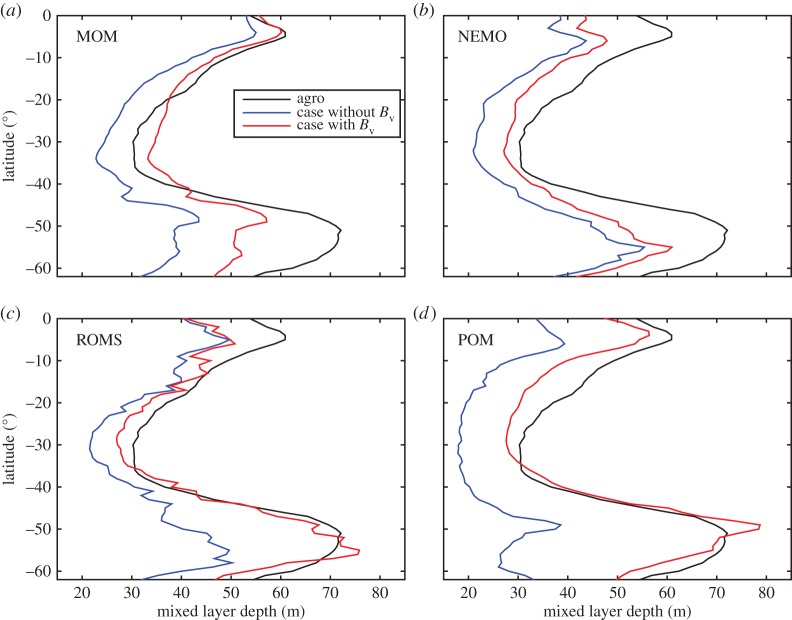
Comparison of the zonal-averaged mixed layer depth in the Southern Ocean in January among Argo data (black), simulation without *B*_v_ (blue) and with *B*_v_ (red) for MOM4 (*a*), NEMO (*b*), ROMS (*c*) and POM (*d*), which indicate the dominant role played by the non-breaking wave-induced vertical mixing in the upper ocean.

The importance of ocean general circulation models (OGCMs) is so high that it cannot be overemphasized. However, most OGCMs, if not all, had suffered from common problems such as the simulated sea surface temperatures are too high, the simulated mixed layer depth (MLD) in the upper ocean is too shallow, or the simulated subsurface temperature is too low in the summer time [42] especially for the Southern Ocean [43], until the non-breaking surface wave-induced vertical mixing was included into OGCMs [6] which much relieves the above-mentioned common problems. In fact, all these challenges indicate that the vertical mixing in the upper ocean has been underestimated. The weak point of the governing equations of all OGCMS is the turbulence closure schemes which have very high uncertainty. Validation of turbulence closure schemes seems a solution to determining which kind of scheme should be selected. However, the only option for turbulence measurements in the open ocean was the indirect dissipation rate [44] before the HHSA is available. The *in situ* observation results [44], which show the dissipation rate decays exponentially in the upper 50 m, confirmed the original parametrization scheme [6]. To show the effects of the non-breaking wave-induced mixing of equation (5.8), here we design four sets of numerical experiments to test the sensitivity of *B*_v_ to different models and turbulence closure schemes. For each set of numerical experiments, the original OGCM was spun up for 10 years, and the model ran with and without *B*_v_ separately for 1 year more. All four OGCMs including MOM4, NEMO, ROMS and POM are popularly used all over the world. MOM4, ROMS and POM employ KPP [5], KPP and Mellor–Yamada [4] turbulence closure schemes, respectively, and the details of model configurations are the same with Shu *et al.* [45], Wang *et al.* [38] and Qiao *et al.* [6]. To reduce the uncertainty of different model configurations, here we use a standard version of NEMO-ORCA1 (http://www.noc.soton.ac.uk/nemo), with coupled ocean and sea-ice configuration based on the ORCA tripolar grid at 1°×1° horizontal resolution and 75 vertical levels, and the atmospheric forcings are standard CORE2. The turbulence closure scheme for NEMO is TKE. There are many different methods and criteria for defining the MLD [46]: the MLD here is defined as the depth where the temperature differs by 0.5° from its value at surface. Although OGCMs and turbulence closure schemes are totally different, all the simulated zonal-averaged MLD are dramatically improved (figure 8), which provide robust evidence that the missed non-breaking wave-induced mixing plays a key role in the upper ocean. The prevailing standpoint that the small-scale ocean surface wave is irrelevant with large-scale ocean circulation is really misleading. The wave-induced vertical mixing can much improve model performance from coastal ocean models [47–49] to global ocean models [50] to climate models [43].

## 6. Conclusion

We have reviewed the past theoretical and experimental studies of wave–turbulence interactions and found that the physics controlling the mechanism of vertical mixing in the ocean surface layer is through the cumulative effect of the wave-induced Stokes drift current. Although this drift is a second-order effect, the overwhelming energy content of the waves makes its second-order effect even more important than the coexisting turbulence. Through the mixing length theory and a dimensional argument, we derived a phenomenological eddy viscosity, which ended up with the identical expression as the one proposed by Yuan *et al*. [35] and Qiao *et al.* [6], which have resulted in drastic improvements of the large-scale ocean surface temperature distribution by removing the system bias in the existing models based on the turbulence closure scheme for mixing. The future expansion of this line of research is to consider the role of internal waves in the deeper layer mixing, which could provide the answer to the inability for most present efforts to model phenomena with periods longer than a decade.

Although wave motions are usually neglected in large-scale motions such as general circulation and climate models, this study has demonstrated that wave motions are of critical importance even in large-scale problems by the role the former play in vertical mixing. The present field experiment has provided direct evidence of wave–turbulence interactions in the field, and also a physical basis for mixing parametrization. Wave effects should definitely be considered in all future large-scale ocean circulation models and climate studies.

## Supplementary Material

Supplementary Material for Wave turbulence interaction induced vertical mixing and its effects in ocean and climate models

## Data accessibility

Data supporting this article are available from the corresponding author (qiaofl@fio.org.cn)
