# Supplementary material for: Wave–turbulence interaction-induced vertical mixing and its effects in ocean and climate models
Source: Philos Trans A Math Phys Eng Sci. 2016 Apr 13;374(2065):20150201. doi: 10.1098/rsta.2015.0201 (PMC4792408; doi:10.1098/rsta.2015.0201)
Supplement: Supplementary Material for Wave turbulence interaction induced vertical mixing and its effects in ocean and climate models [file rsta20150201supp1.docx]

# Supplementary Material for

# Wave turbulence interaction induced vertical mixing and its effects in ocean and climate models

by

Fangli Qiao^1,2,3^, Yeli Yuan^1^, Jia Deng^1^, Dejun Dai^1^ and Zhenya Song^1^

1. First Institute of Oceanography, State Oceanic Administration, Qingdao, China, 266061
2. Laboratory for Regional Oceanography and Numerical Modeling, Qingdao National Laboratory for Marine Science and Technology, Qingdao, China, 266071
3. Key Lab of Marine Science and Numerical Modeling, SOA, Qingdao 266061, China

# The experimental setup

# As shown in the paper, three ADVs were attached to OPMM at vertical intervals of 1.5m, with the upper ADV at 2.6 m below the mean water level. The data are stored in a recorder encased in the instrument. As recording cables are connected with all the three ADVs, any changes in the experiment together with the observed data could be saved to the computer. Soon after the instruments were installed, Rammasun, a category 4 typhoon passed by, inflicting serious damage: although the middle and lower ADVs stay connected with the steel frame, the upper ADV was lost.

# Fortunately, observed data from the three ADVs within the first 24 hours, i.e., from 17:00 July13 to 16:30 July 14 had been saved to the computer before Rammasun started to affect the South China Sea. As a result, only 24 hours data were obtained for the upper ADV, which was eventually lost. For the middle and lower ADVs, the recovered recorder covered for about 185 hours, from 17:00 July 13 to 10:00, July 21, but only the data for the initial 108 hours, lasting from 17:00 July 13 to 03:30, July 18 are amenable to further analysis because, from 03:30 July 18 onwards (Figure S6), the heading, pitch, and roll showed that the frame attached ADVs with OPMM began to waggle due to the effect of Rammasun. Consequently, only data collected by the lower ADV with high sampling frequency are used in this paper;

# Besides the three ADVs, bottom supported ADV and AWAC (Acoustical Wave and Current, Nortek), were arranged about 100m away from the OPMM, in order to observe current profile and surface waves (Figure S1). Operating under the constraint of having to cover the entire experimental period with limited memory, measurement of the bottom ADV was made at 30-minute intervals for 3 minutes duration at the sampling rate of 8Hz. For current measurement of AWAC, the vertical interval was set to 1.0m at 1800s burst intervals, with mean current velocity and direction measurements recorded continuously for 180s. For surface wave measurement of AWAC, the burst interval was also set at 1800s while the observing duration was 1024s. Impacted by Rammasun, the whole bottom supported buoy turned over at 10:00 July 18. Thus only the data for current and surface waves covered from 17:00 July 13 to 09:30 July 18 are useful.

# Traditional wind speed and direction were observed on the OPMM. In addition, a meteorological and marine buoy, about 100 km away from OPMM, hoisted by Guangzhou Institute of Tropical and Marine Meteorology, China Meteorology Administration, operationally recorded the wind and surface waves (Figure S1). 10 min-mean wind speed, wind direction and 10 min-mean significant wave height and period were provided by Guangzhou Institute of Tropical and Marine Meteorology.

# Weather charts and long term data showing how sea states changed as typhoon Rammasun approached.

The weather charts, based on ocean-atmospheric coupled model results with data assimilation, are shown in Figure S2, from which we can see that Rammasun, a category 4 typhoon influenced South China Sea from 16 July and made a landfall over the nearby Hainan Island, on 18 July 2014. Wind speed and direction observed by anemometers on the buoy and OPMM were shown in Figure S3 and S4. The maximum 10-min mean wind speed (15m above water surface) approached 27m/s, as shown in Figure S3.The significant wave height approached 9.7m (Figure S3), as observed by the meteorology and marine buoy while the maximum significant wave height was 3.4m near OPMM (Figure S4). The red arrows in Figure S3 and S4 denote the time 15:00, 15 July (well ahead of the typhoon) and 17:30, 17 July (just before the instrument was damaged) when the data selected to be analyzed in the paper were obtained. At the two recording times, the observed significant wave heights by AWAC were 0.8m and 1.9m, respectively.

Evolution of current speed and direction were shown in Figure S5. Tidal current dominates in the shallow area. At 15:00, 15 July, the vertical averaged current speed was 23cm/s with eastward direction while at 17:30, 17 July, it was 8cm/s with south-eastward direction.

Figure S6 shows the heading, mean depth of each burst, and residual depth observed by lower ADV. The blue arrow in the subfigure of heading denotes 03:30, July 18. At this moment the frame attached ADVs with OPMM began to waggle. As Rammasun passed by, the waggling intensified. At 16:30, July 18, the solid connection between the frame and OPMM was suspected to be broken and only kevlar cables protected the frame with ADVs not leaving OPMM, which could be found in abrupt change of mean depth (Figure S6b). The mean depth before 03:30, July 18, can characterize the sea level variation induced by tide. The maximum tidal range during that time was about 3.3m (Figure S6b). The residual depth is the 65 points running mean of the original observed depth minus the mean depth. The residual depth can be considered as, but not absolute, the surface elevation induced by surface waves. The red arrows denote the time 15:00, 15 July (well ahead of the typhoon) and 17:30, 17 July (just before the instrument was damaged) when the data selected to be analyzed in the paper were obtained.

# Data cleaning method and the whole series of results for the horizontal components for Sections 1 and 2.

Variations of mean signal to noise ratio (snr) and mean correlation of lower ADV were shown in Figure S7. Snr and correlation were directly output from the instrument, which were usually used to examine data quality. From the figure, we can see that both snr and correlation show variation with time, which was related with tidal cycle. The possible reason is that spring tide carried clean water from deep ocean while ebb time brought near shore water with large amounts of particles to the station The particles serve to influence the quality of ADV data. In our experiments, the snr and correlation were used to check the data quality. For every burst, the data would be considered as low-quality data if the number of data with snr less than 5 or correlation less than 60 was larger than 30% of the whole data length. Otherwise the data is acceptable. Further analysis and deep investigation were carried out for the high-quality data while the low-quality data was withdrawn.

Two de-spike processes were carried out to remove the outliers. For the first de-spike process (Figure S8), 65 point running mean was used to obtain the smoothed data from the original observed data. The residual data was obtained by original data minus smoothed data. The residual data, larger than 3 standard deviations (STD), were selected and replaced by white noise with STD equal to that of the residual data. The new residual data plus the smoothed data was the data after first de-spike process. The second process is the same as the first de-spike, but the data after first de-spike was used as the original data. After the two de-spike processes, almost all the spikes can be removed.

The HHSA here is carried out on the cleansed data, i.e., the data after two de-spike processes. We present the results for horizontal components of Data Section 1 and 2 in Figures S10 to S21. As was done for vertical component, raw data have been cleaned by removing some occasional outliers for the first step. Since IMF component 1 generally cannot pass the significance test, hence, we no longer show the Holo-Hilbert spectra and phase distribution for this component in the following figures to save space.

# First consider u component of Data Section 1 in Figures S10 to S12. From Figure S10a, where Fourier spectra for raw and cleansed data are given, there exists a prominent spectral peak near 0.2 Hz, and whereas, the Kolmogorov -5/3 power law is nearly indistinguishable except for a very narrow range around 1Hz. In Figure S10b, cleansed data is decomposed into 12 IMFs plus a trend, amongst which IMF 1 to 5 are white noise and wave components are concentrated in IMF 6, 7 according to the significance test in Figure S10c. Blue line in Figure S11 is the extracted wave by adding IMF 6, 7 with trend together, green line represents raw data, red line is residue after removal of wave, and black line in the lower panel is higher frequency obtained by filtering lower frequency than wave from red line. Figure S10d is the probability test for red and black lines in Figure S11. The sum of higher frequency components (i.e., black line being the sum of IMF number 1 to 5) shows a distinct non-Gaussian characteristics with skewness at 0.02 and kurtosis at 3.67 instead of 3.00. While, the full residue contains all motions but surface wave also exhibits a non-Gaussian distribution with skewness at -0.08 and kurtosis at 3.48.

# Figure S12 show the results of HHSA and phase distribution for IMF components 2 to 5, where Figure S12a is for IMF2 with a frequency range of 6 to 14 Hz, Figure S12b is for IMF3 with a frequency range of 4 to 6 Hz, Figure S12c is for IMF4 with a frequency range of 2 to 3 Hz, and Figure S12d is for IMF 5 with a frequency range of 0.5 to 1.5 Hz. Considering both significance test and phase distribution, these IMF components are more likely to be white noise. Hence, the phenomenon of a crest-locked or trough-locked AM modulation of turbulence is insignificant in this group of data.

# The corresponding results for v component of Data Section 1 are given in Figures S13 to S15. From Fourier spectra in Figure S13a, there is hardly a range for the establishment of the Kolmogorov -5/3 power law. Therefore, the subsequent analysis in Figures S13b to S15 makes little sense.

# Next consider u component of Data Section 2 in Figures S16 to S18. From these figures, the Kolmogorov -5/3 power law is visible in the range of 0.6 to nearly 1Hz. The IMF and significance test indicate that IMF 5 may contain some turbulence and the energy density increased drastically from IMF 6. For the probability density, the sum of high frequency components obviously deviates from a Gaussian distribution with skewness at 0.02 and kurtosis at 4.63. The full residue has skewness at 0.11 and kurtosis at 3.23. The HHSA results are given in Figure S18, where a visible crest-locked AM modulation of the turbulence is revealed in Figure S18d.

# Finally, the results for v component of Data Section 2 are given in Figures S19 to S21. According to S19a, the Kolmogorov -5/3 power law may be valid in a very narrow band around 1Hz. While, the significance test suggests that IMF 1 to 5 are white noise and IMF 6 to 8 contain the information of wave motions. Besides, the phase distribution in Figure S21 together with Fourier spectra also enhance that IMF 1 to 5are indeed noise. As a conclusion, it does not suffice for a crest-locked or trough-locked AM modulation of turbulence with this group of data.

# The Phase-locking Mechanism of Nonlinear Wave Modulation

It is interesting to note that most actual modulations between surface wave and the turbulence are always locked at the trough in the field in our experiment and in the laboratory (Thais and Magnaudet, 1996). The phase of wave turbulence modulation should be symmetric if the modulating wave is pure sinusoidal. What could be the cause of this asymmetry? Thais and Magnaudet (1996) have suggested that the non-sinusoidal properties of modulating wave might be responsible, but no direct evidence was presented. To clarify the mechanism of this preferred phase locking phenomena, we have made a study with a simple model to demonstrate that the cause is exactly the nonlinear distortion of modulating waves. Three kinds of modulating waves are generated to demonstrate our claim: a pure sine wave and two nonlinear distorted waves, all modeled by

$w(t)=\cos\left(\omega t\pm\varepsilon\sin\omega t\right)$ . (s1)

When ***ε =0***, we have the pure sine wave; otherwise, we have the asymmetric Stoke type of waves. The full data are given by

$x(t)=w(t)+w(t)\sin\left(2\pi t\right)\text{ ; with }\omega\text{=}\frac{10}{16}\text{ and }\varepsilon\text{=0 or 0.5}$. (s2)

The simulated results are given in Figures S22 to S24. Figure S22a gives the data for pure sine waves, and Figure S22b gives Holo-Hilbert spectrum and phase locking distribution. Clearly, phase distribution is symmetric, which means modulation occurs at both the trough and the peak equally. As a result, modulation should occur at twice the frequency of modulating waves, at near 0.2 Hz as shown in Holo-Hilbert spectrum. When we take plus sign in Equation (s1) and set ***ε =0.5***, we have data and Holo-Hilbert spectrum given in Figures S23a and b. Now phase distribution is locked only in the trough region, and modulating frequency should be the same frequency of modulating waves at near 0.1 Hz. If we take minus sign in Equation (s1) and set ***ε =0.5*** again, we have data and Holo-Hilbert spectrum given in Figure S24a and b. Now phase distribution is locked only in the peak region, and modulating frequency again is also at the frequency of modulating waves, near 0.1 Hz. Thus, it is clear that the asymmetric phase lock mechanism is due to the nonlinearity of modulating waves. The stronger the nonlinearity, the stronger the asymmetric distribution. This is exactly what we have observed.

**Figure captions for supplement materials**

Figure S1. The experiment site and the ancillary measurements include bottom supported ADV and AWAC (Acoustical Wave and Current), and a meteorology buoy about 100km away from OPMM.

Figure S2. Weather charts from July 13 to 18, 2014. The weather charts were obtained from ocean-atmosphere coupled model results with data assimilation.

Figure S3. Evolution of 10min-mean wind (15m above water surface) and 10min-mean significant height and period of surface waves observed by the meteorology and marine buoy from 17:00, July 13 to 7:10, July 19. The red arrows denote the time 15:00, July 15 and 17:30, July 17 when the data selected to be analyzed in the paper were obtained.

Figure S4. Evolution of 10min-mean wind (13m above mean water surface) observed by anemometer on OPMM and significant height and period of surface waves observed by bottom supported AWAC near OPMM during the experiments. The red arrows denote the time 15:00, July 15 and 17:30, July 17 when the data selected to be analyzed in the paper were obtained.

Figure S5. Evolution of current observed by bottom supported AWAC near OPMM during the experiments. The black arrows denote the time 15:00, July 15 and 17:30, July 17 when the data selected to be analyzed in the paper were obtained.

Figure S6. Evolution of heading, mean depth of each burst, residual depth observed by lower ADV where mean depth denotes the depth averaged over each burst, i.e., 180s while residual depth is the 65 points running mean of the original observed depth minus the mean depth. The red arrows denote the time 15:00, July 15 and 17:30, July 17 when the data selected to be analyzed in the paper were obtained. The blue arrow in the subfigure of heading denotes 03:30, July 18 after this time the frame attached ADVs with OPMM began to waggle.

Figure S7. Evolution of mean signal to noise ratio (snr) and correlation output from lower ADV. The red arrows denote the time 15:00, July 15 and 17:30, July 17 when the data selected to be analyzed in the paper were obtained.

Figure S8. First de-spike process of the vertical component of velocity measurements at 15:00, July 15.

Figure S9. Second de-spike process of the vertical component of velocity measurements at 15:00, July 15.

Figure S10 (a) to (d).

(a). Various Fourier spectra of u component, raw and cleansed data, the part for waves and residue after wave motions have been removed through EMD of Data Section 1 collected at 15:00, 15 July 2014, ahead of Typhoon.

(b). The IMFs of cleansed data: wave motions can be seen in components 6 and 7.

(c). Significance test of u component of Data Section 1: Components 1 to 5 have the same characteristics as white noise; components 6 and 7 are the most energetic wave motions; the rest is for large scale motions.

(d). Probability distribution density of various data components: both residue (thin solid line) containing all motions except waves and high frequency turbulence containing noise (thick solid line) are non-Gaussian. The Gaussian model is given in the dotted line.

Figure 11. Data and separation of waves and high frequency component: cleansed data (light green) and wave motions (blue) reconstituted by components 6, 7 and final trend to give it on the right level; residue (red) defined as the difference between data and wave motions; high frequency component may contain turbulence given in the lower panel.

Figure S12(a) to (d).

The Holo-Hilbert spectral analysis and phase locking test for u component of Data Section 1.

(a). The Holo-Hilbert spectrum of IMF 2 (upper panel) and phase distribution (lower panel) with gray zone indicating ± standard deviation confidence bound: notice that this component should be white noise based on Fourier spectral analysis, significance test, and nearly uniform phase distribution.

(b). Same as in Figure S12(a): the component of IMF 3 is indeed noise.

(c). Same as in Figure S12(a): the component of IMF 4 is indeed noise.

(d). Same as in Figure S12(a): the component of IMF 5 is indeed noise.

Figure S13(a) to (d).

(a). Same as in Figure S10(a): here data is v component of Data Section 1.

(b). Same as in Figure S10(b): wave motions can be seen in components 6 and 7.

(c). Same as in Figure S10(c): here significance test also indicates that the components 6 and 7 are energetic and information containing.

(d). Same as in Figure S10(d): both residue (thin solid line) containing all motions except waves and high frequency turbulence containing noise (thick solid line) are non-Gaussian. The Gaussian model is given in the dotted line.

Figure S14. Same as in Figure S11: cleansed data (light green) and wave motions (blue) reconstituted by components 6, 7 and final trend to give it on the right level; residue (red) defined as the difference between data and wave motions; high frequency component may contain turbulence given in the lower panel.

Figure S15(a) to (d).

The Holo-Hilbert spectral analysis and phase locking test for v component of Data Section 1.

(a). Same as in Figure S12(a): the component of IMF 2 is indeed noise.

(b). Same as in Figure S12(a): the component of IMF 3 is indeed noise.

(c). Same as in Figure S12(a): the component of IMF 4 is indeed noise.

(d). Same as in Figure S12(a): the component of IMF 5 is indeed noise.

Figure S16(a) to (d)_.

(a). Same as in Figure S10(a), here data is u component of Data Section 2, when the experiment site is under the influence of Typhoon Rammasun. Notice the broadening and down shift of the wave energy in Fourier spectrum.

(b). Same as in Figure S10(b): wave motions can be seen in components 6 and 7.

(c). Same as in Figure S10(c): components 1 to 4 have the same characteristics as white noise; component 5 is beyond white noise and should contain turbulence; components 6 and 7 are the most energetic wave motions; the rest is for large scale motions.

(d). Same as in Figure S10(d): both residue (thin solid line) containing all motions except waves and high frequency turbulence containing noise (thick solid line) are non-Gaussian. The Gaussian model is given in the dotted line.

Figure S17. Same as in Figure S11: cleansed data (light green) and wave motions (blue) reconstituted by components 6, 7 and trend to give it on the right level; residue (red) defined as the difference between data and wave motions; high frequency component may contain turbulence given in the lower panel.

Figure S18(a) to (d).

The Holo-Hilbert spectral analysis and phase locking test for u component of Data Section 2.

(a). Same as in Figure S12(a): the component of IMF 2 is indeed noise.

(b). Same as in Figure S12(a): the component of IMF 3 is indeed noise.

(c). Same as in Figure S12(a): the component of IMF 4 is indeed noise.

(d). Same as in Figure S12(a): here IMF component 5 contains genuine information, and phase distribution is slightly shifted toward to the frontal part of wave.

Figure S19(a) to (d).

(a). Same as in Figure S10(a), here data is v component of Data Section 2.

(b). Same as in Figure S10(b): wave motions can be seen in components 6, 7 and 8.

(c). Same as in Figure S10(c): components 1 to 5 have the same characteristics as white noise; components 6, 7 and 8 are the most energetic wave motions; the rest is for large scale motions.

(d). Same as in Figure S10(d): both residue (thin solid line) containing all motions except waves and high frequency turbulence containing noise (thick solid line) are non-Gaussian. The Gaussian model is given in the dotted line.

Figure S20. Same as in Figure S11: cleansed data (light green) and wave motions (blue) reconstituted by components 6, 7, 8 and trend to give it on the right level; residue (red) defined as the difference between data and wave motions; high frequency component may contain turbulence given in the lower panel.

Figure S21(a) to (d).

The Holo-Hilbert spectral analysis and phase locking test for v component of Data Section 2.

(a). Same as in Figure S12(a): the component of IMF 2 is indeed noise.

(b). Same as in Figure S12(a): the component of IMF 3 is indeed noise.

(c). Same as in Figure S12(a): the component of IMF 4 is indeed noise.

(d). Same as in Figure S12(a): the component of IMF 5 is indeed noise.

Figure S22 (a) to (b). A simple model to illustrate phase locking mechanism of modulated waves with respect to modulating waves due to the nonlinear distortion of modulating wave profiles.

(a). The model data of pure sine modulating wave (upper panel) and the full surface time series.

(b). The Holo-Hilbert spectrum (upper panel) indicating a 1 Hz wave is modulated by a 0.1 Hz symmetric pure sine wave. The phase distribution appears both at the peak and trough regions modulated at twice the modulating frequency.

Figure S23 (a) to (b). Same as Figure S22 with

(a). The model data of a Stokes modulating wave (upper panel) and the full surface time series.

(b). The Holo-Hilbert spectrum (upper panel) indicating a 1 Hz wave is modulated by a 0.1 Hz asymmetric Stokes type wave. The phase distribution is locked in the trough regions modulated at the modulating frequency.

Figure S24(a) to (b). Same as Figure S22 with

(a). The model data of an up-side-down Stokes modulating wave (upper panel) and the full surface time series.

(b). The Holo-Hilbert spectrum (upper panel) indicating a 1 Hz wave is modulated by a 0.1 Hz up-side-down asymmetric Stokes type wave. The phase distribution is locked in the peak regions modulated at the modulating frequency.


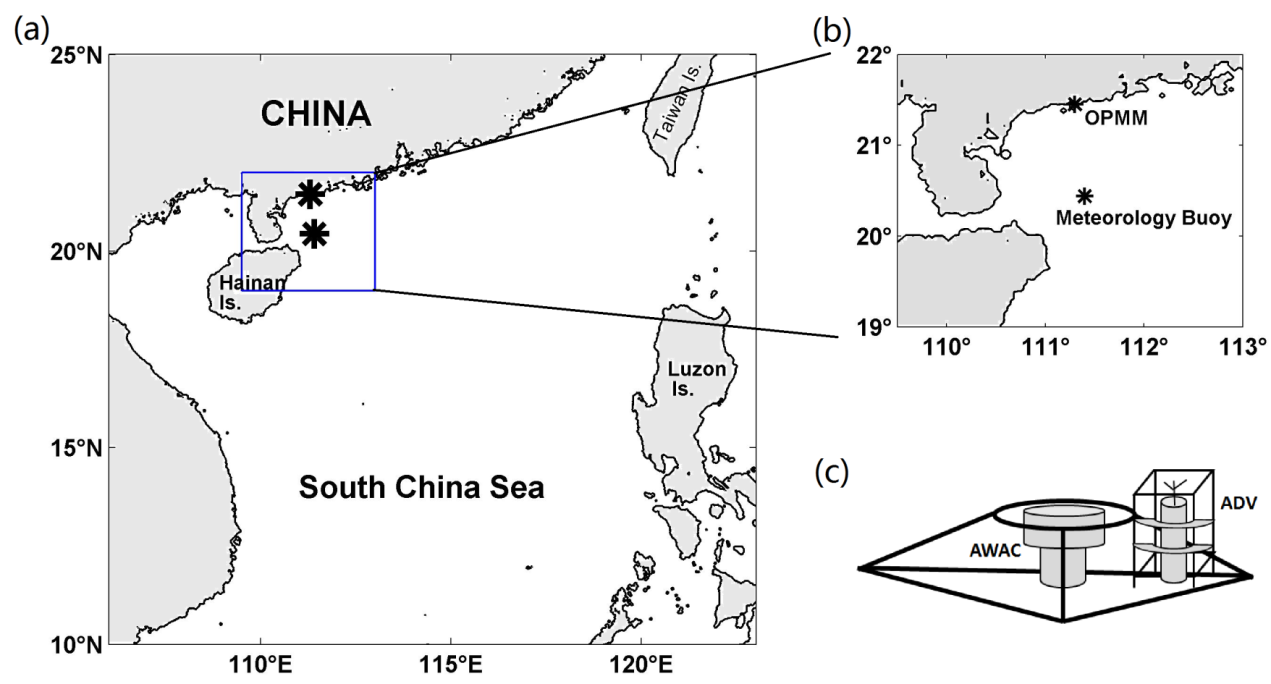


Fig. S1


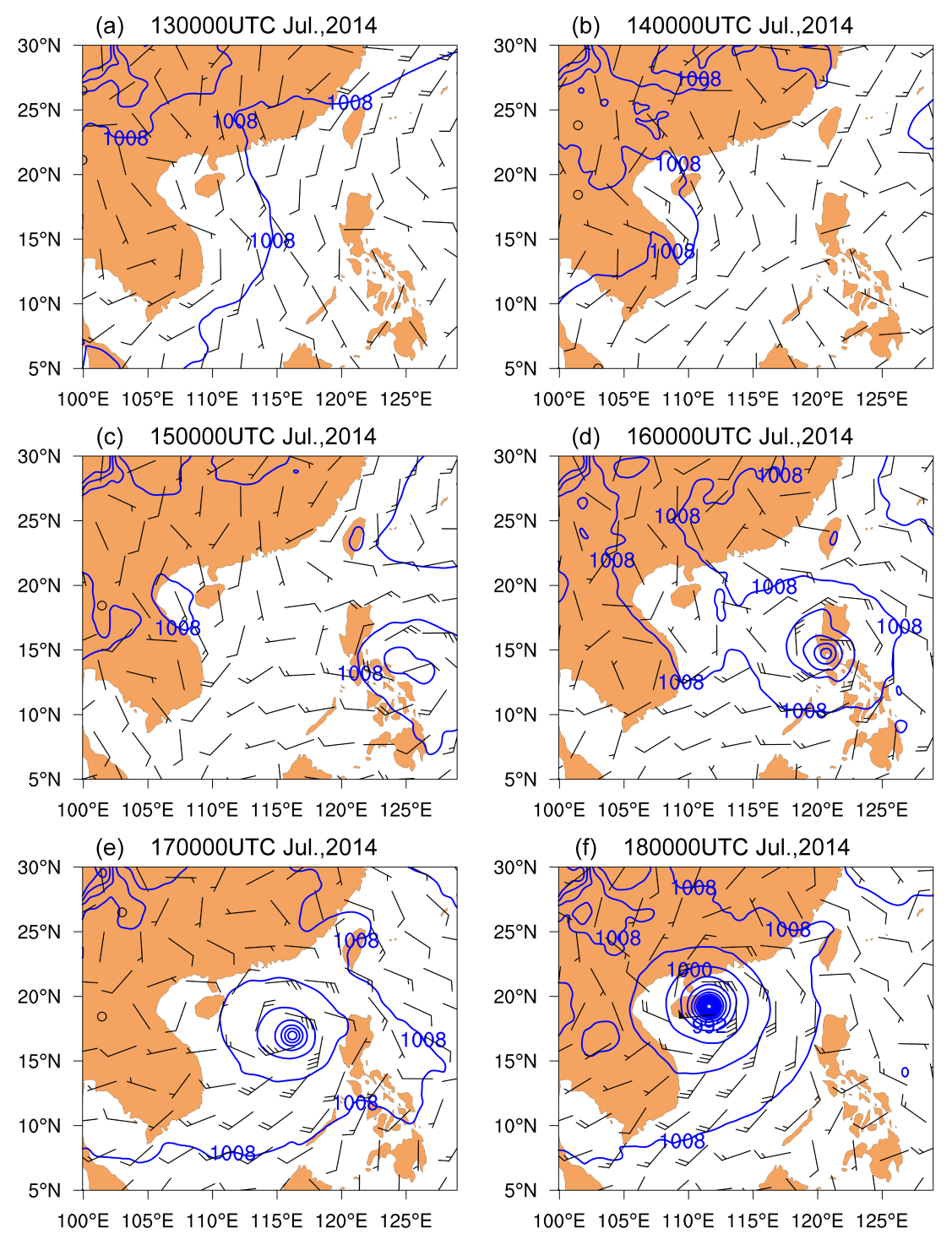


Fig. S2


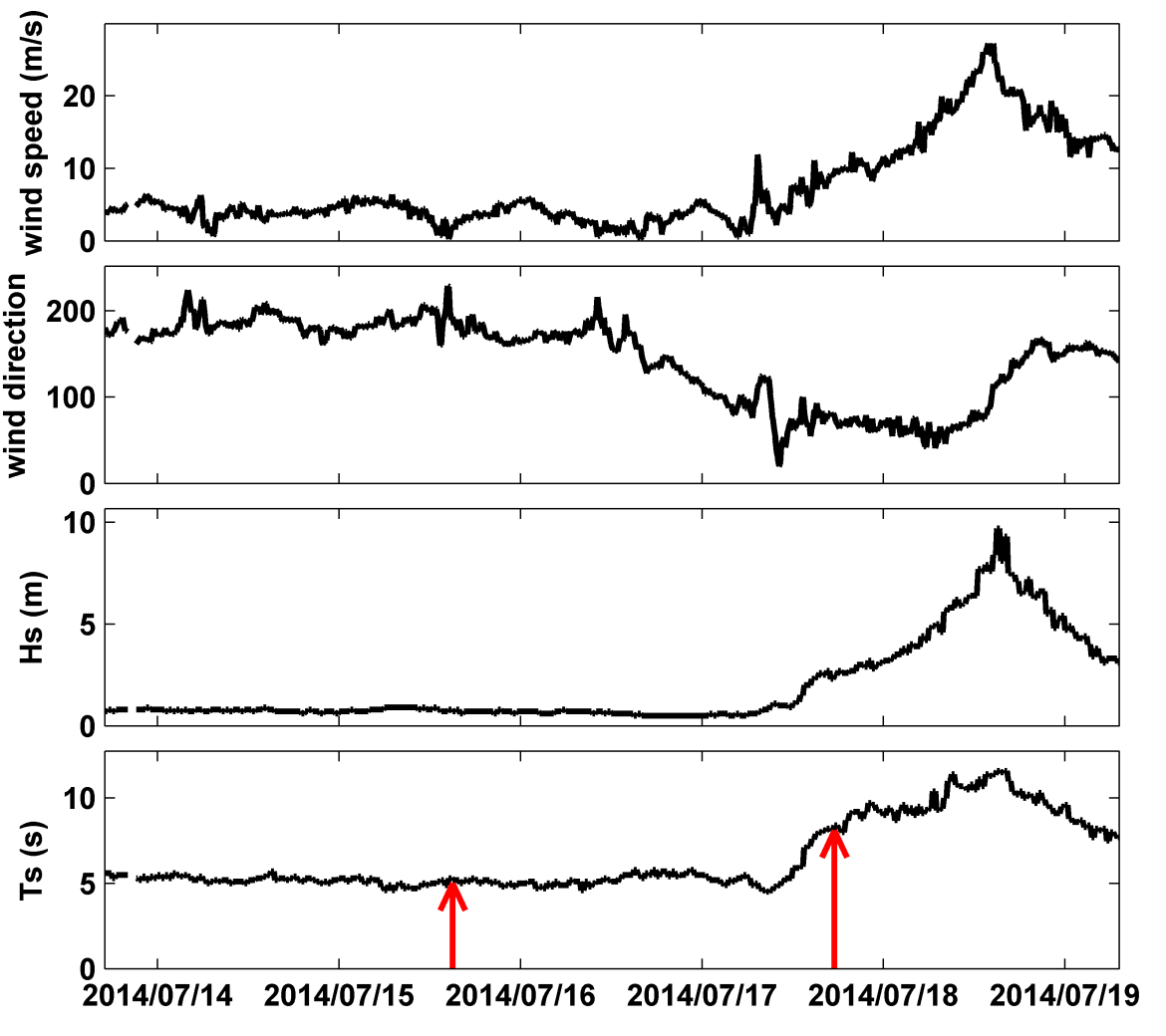


Fig. S3


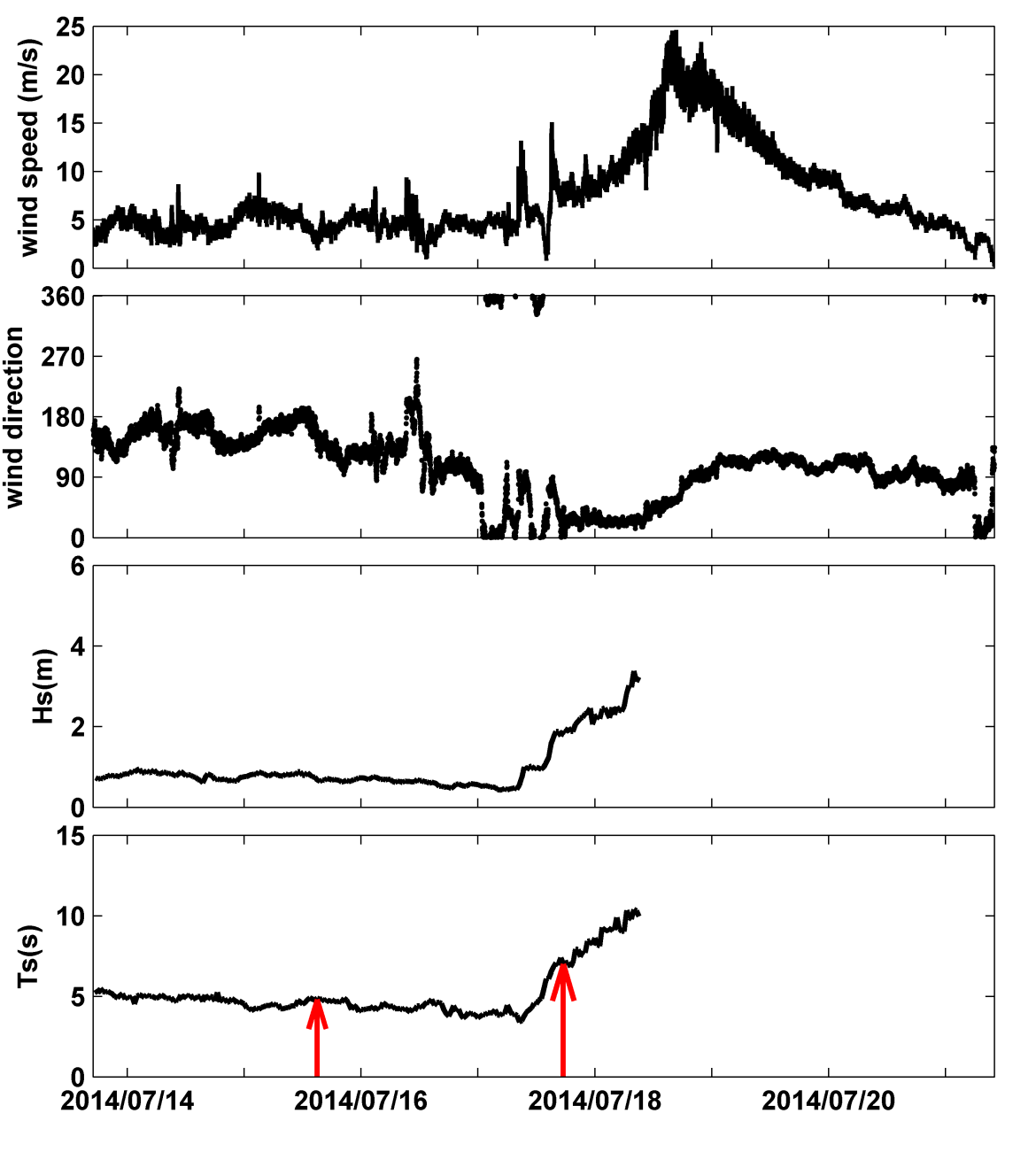


Fig. S4


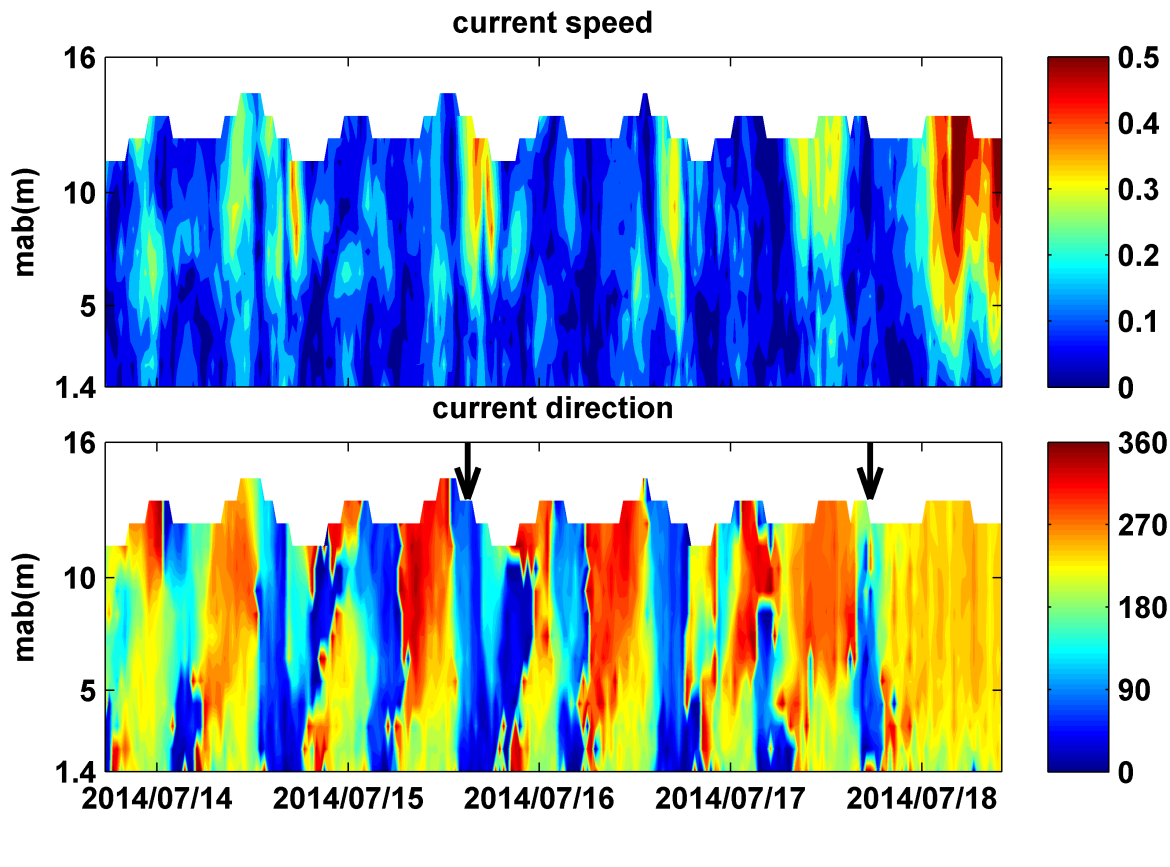


Fig. S5


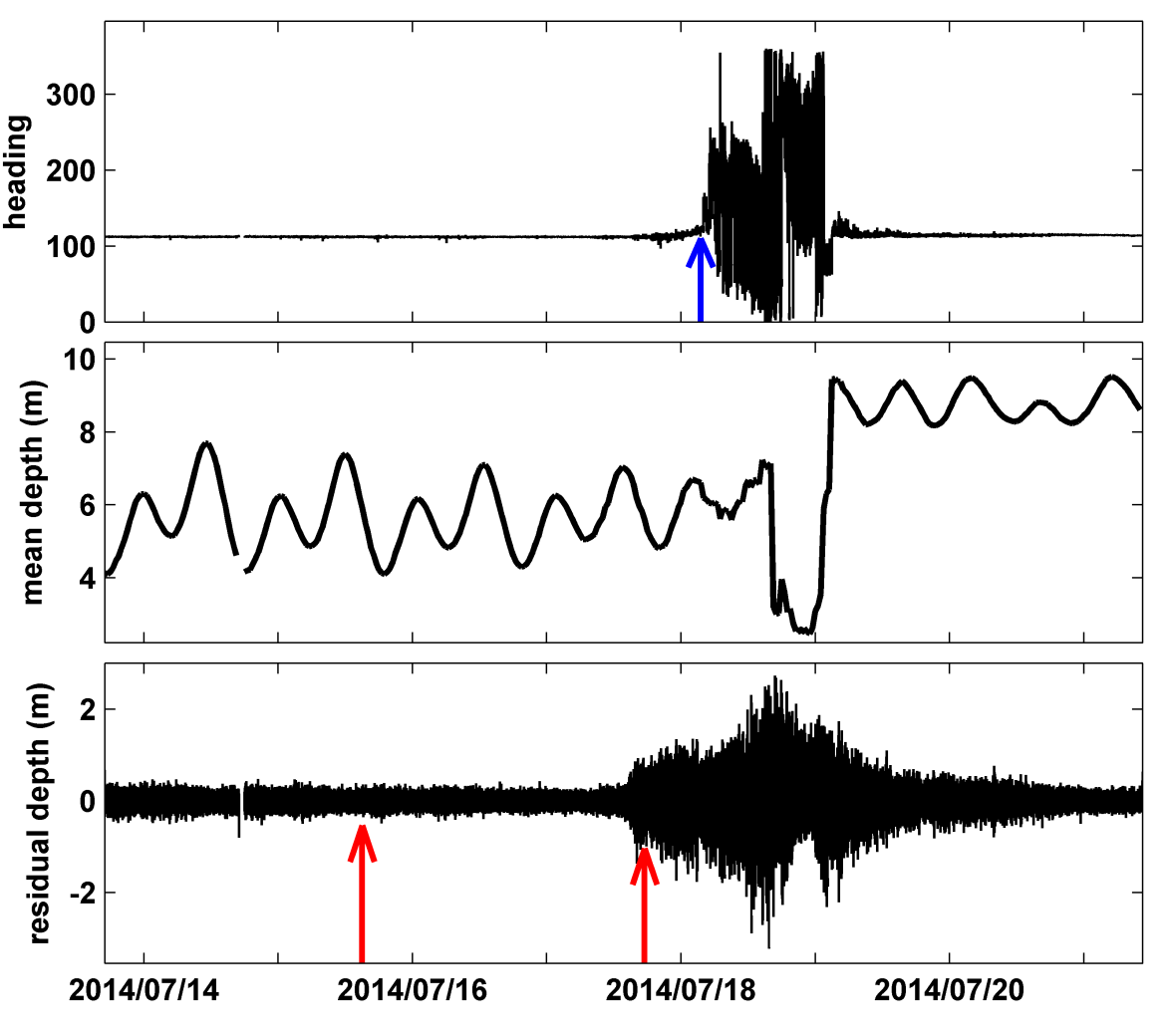


Fig. S6


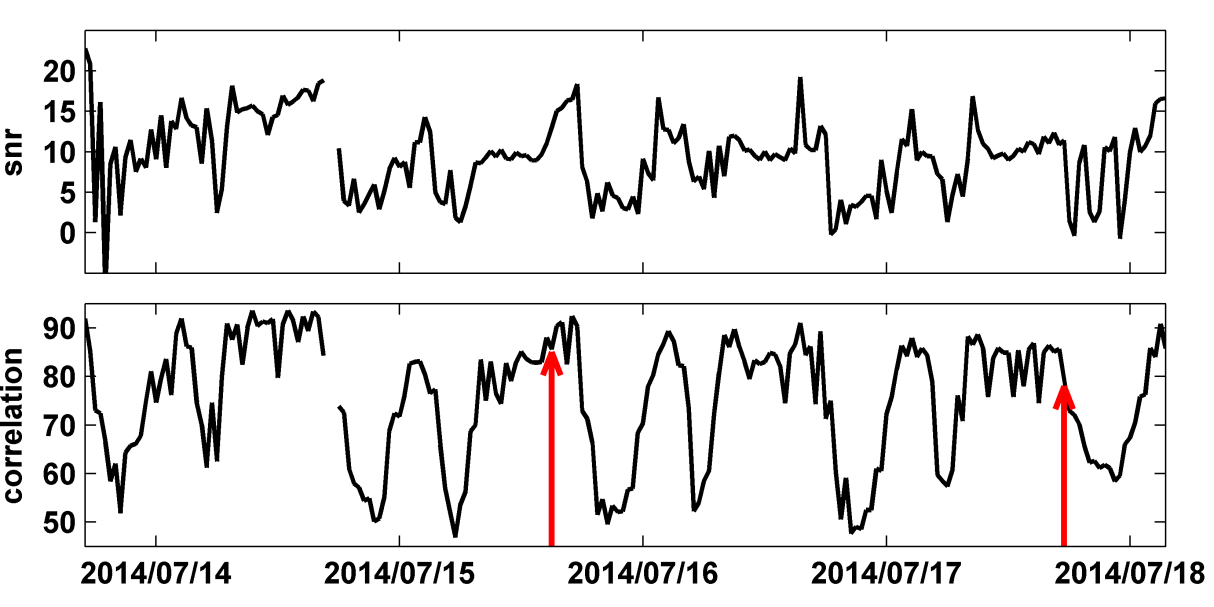


Fig. S7


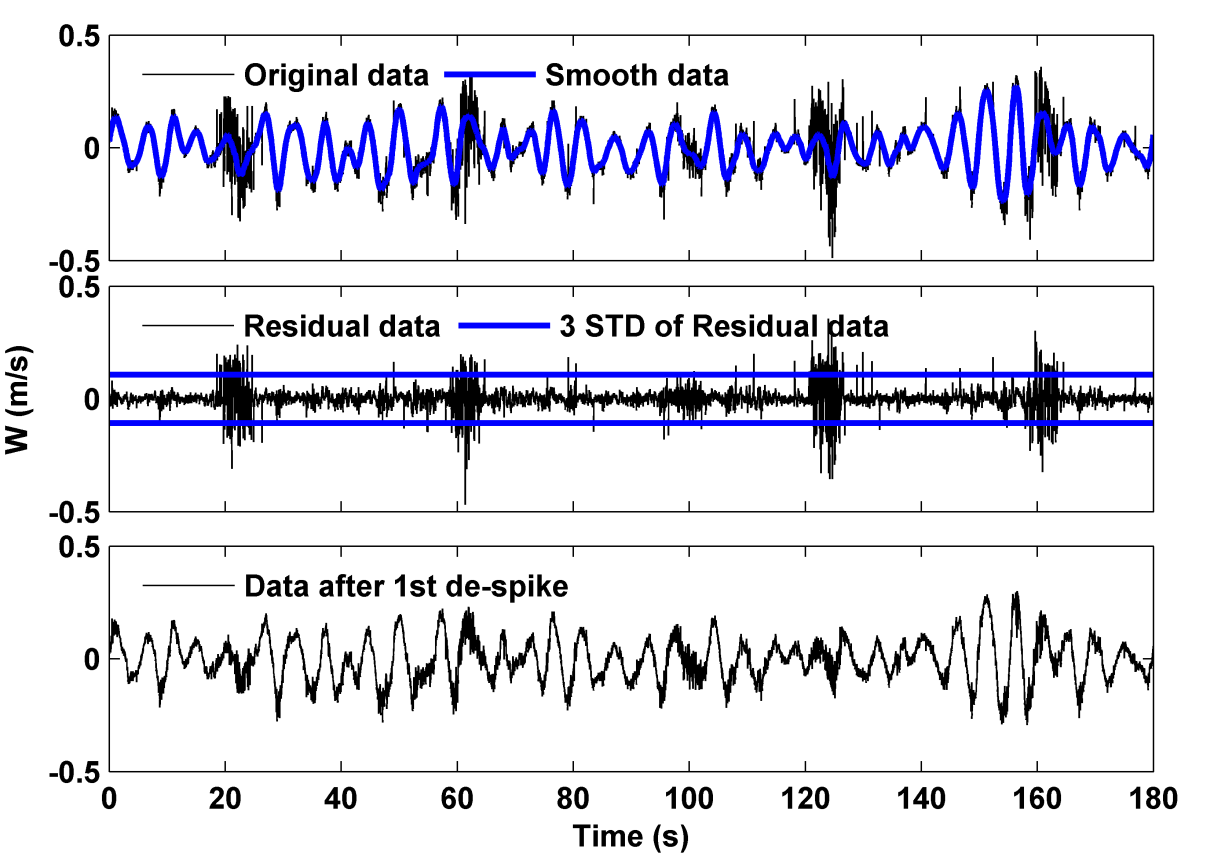


Fig. S8


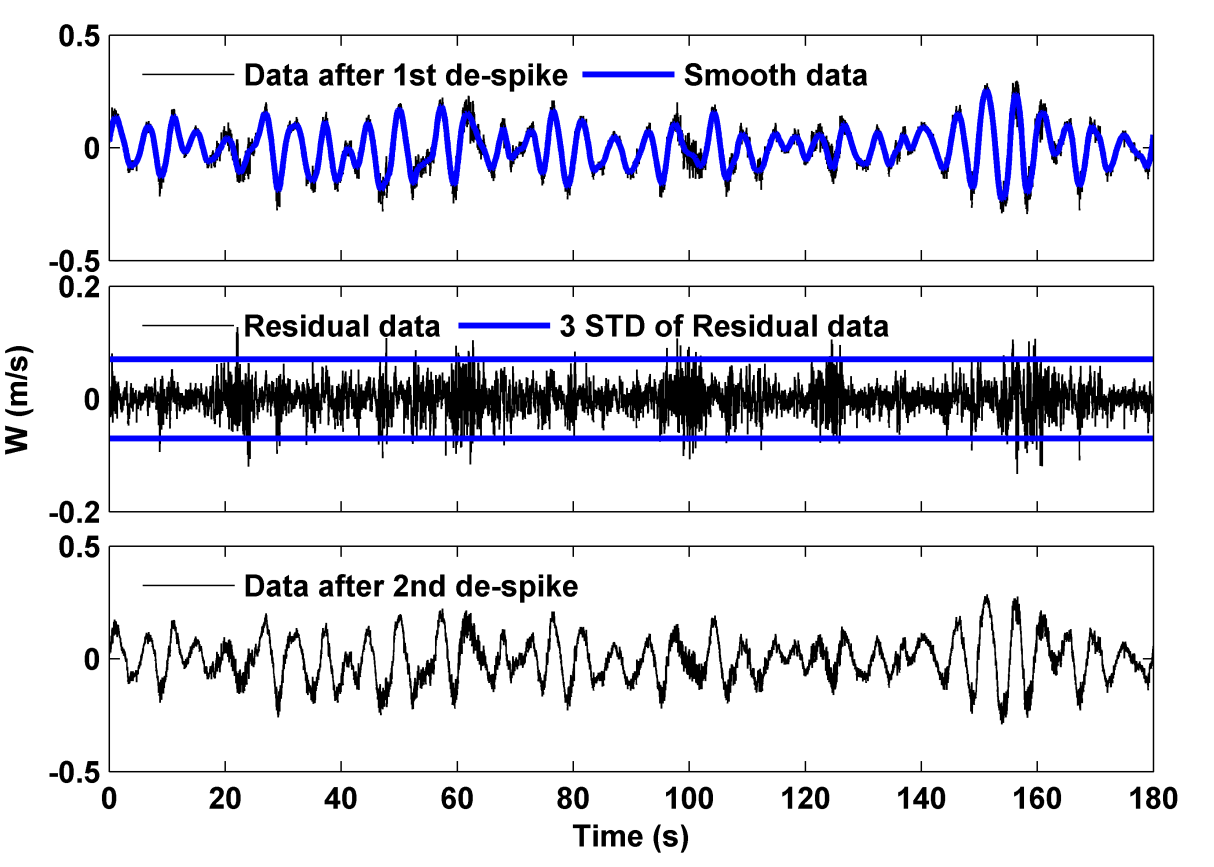


Fig. S9


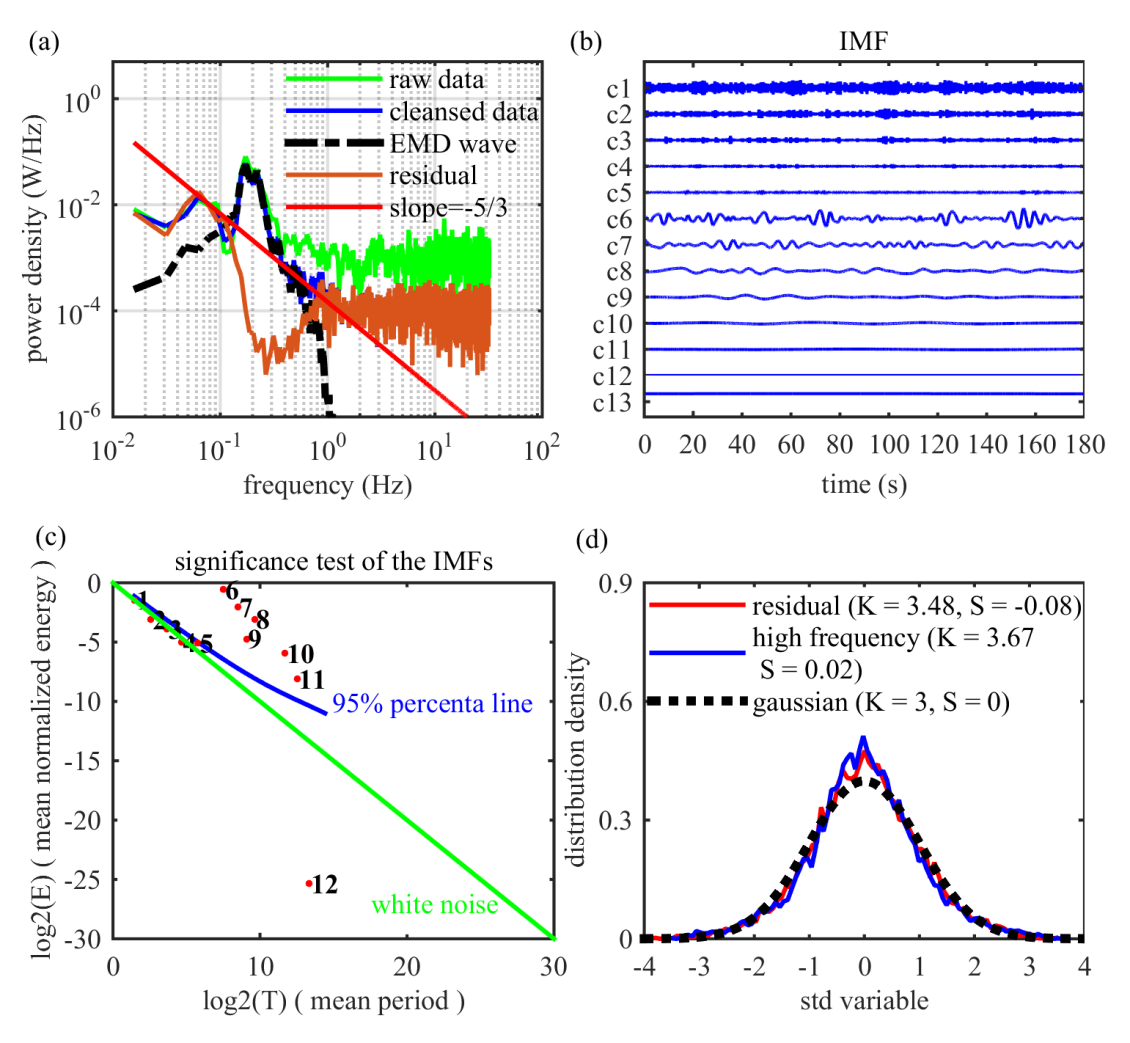


Fig. S10


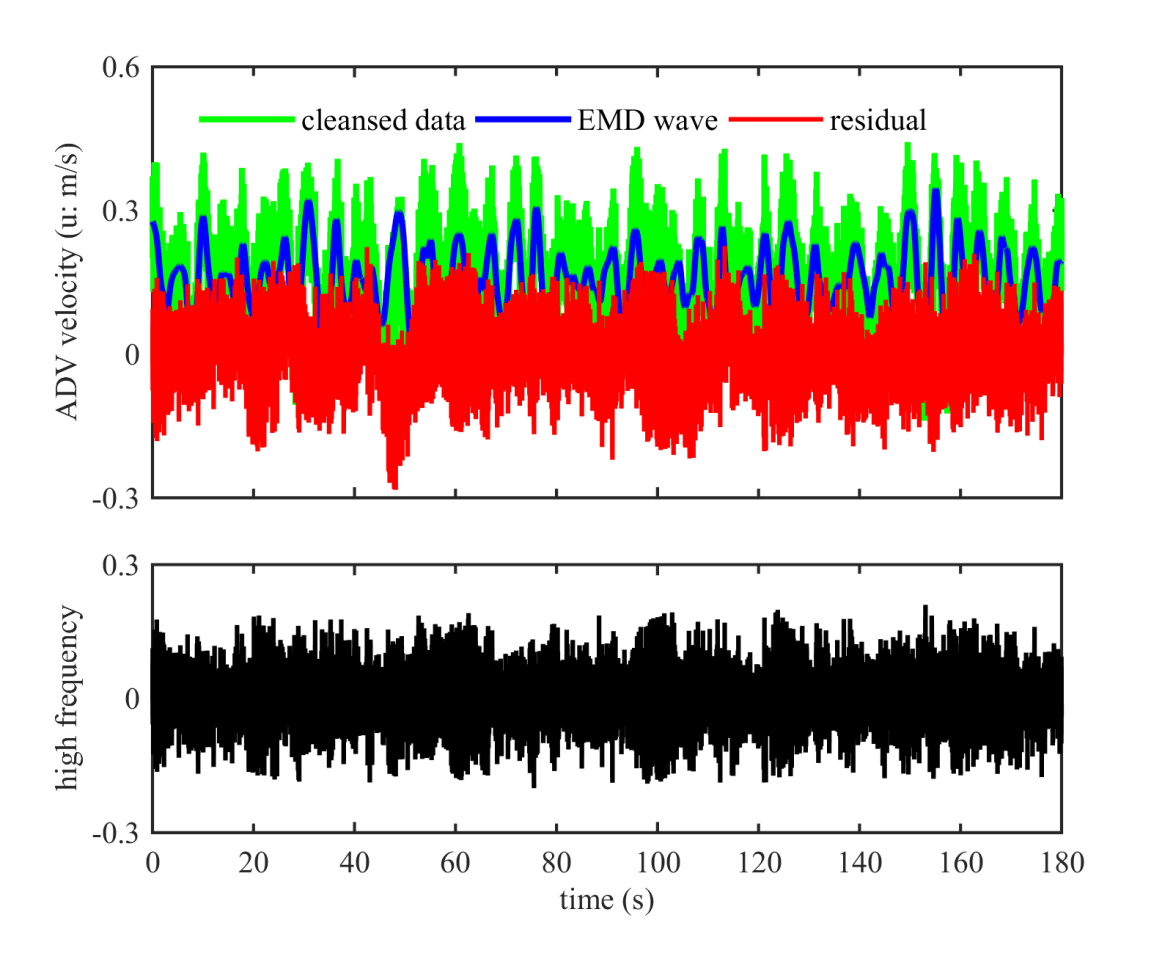


Fig. S11


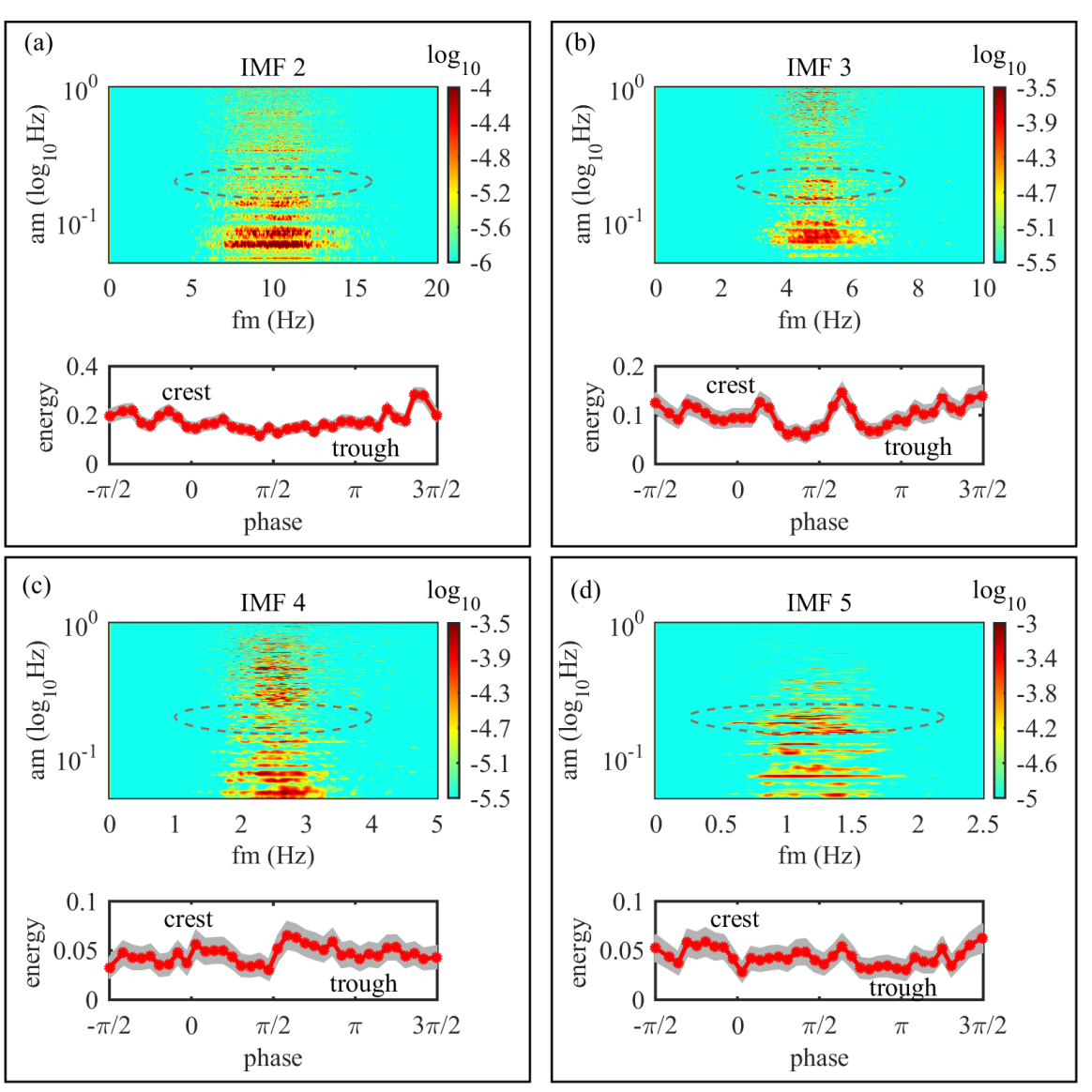


Fig. S12


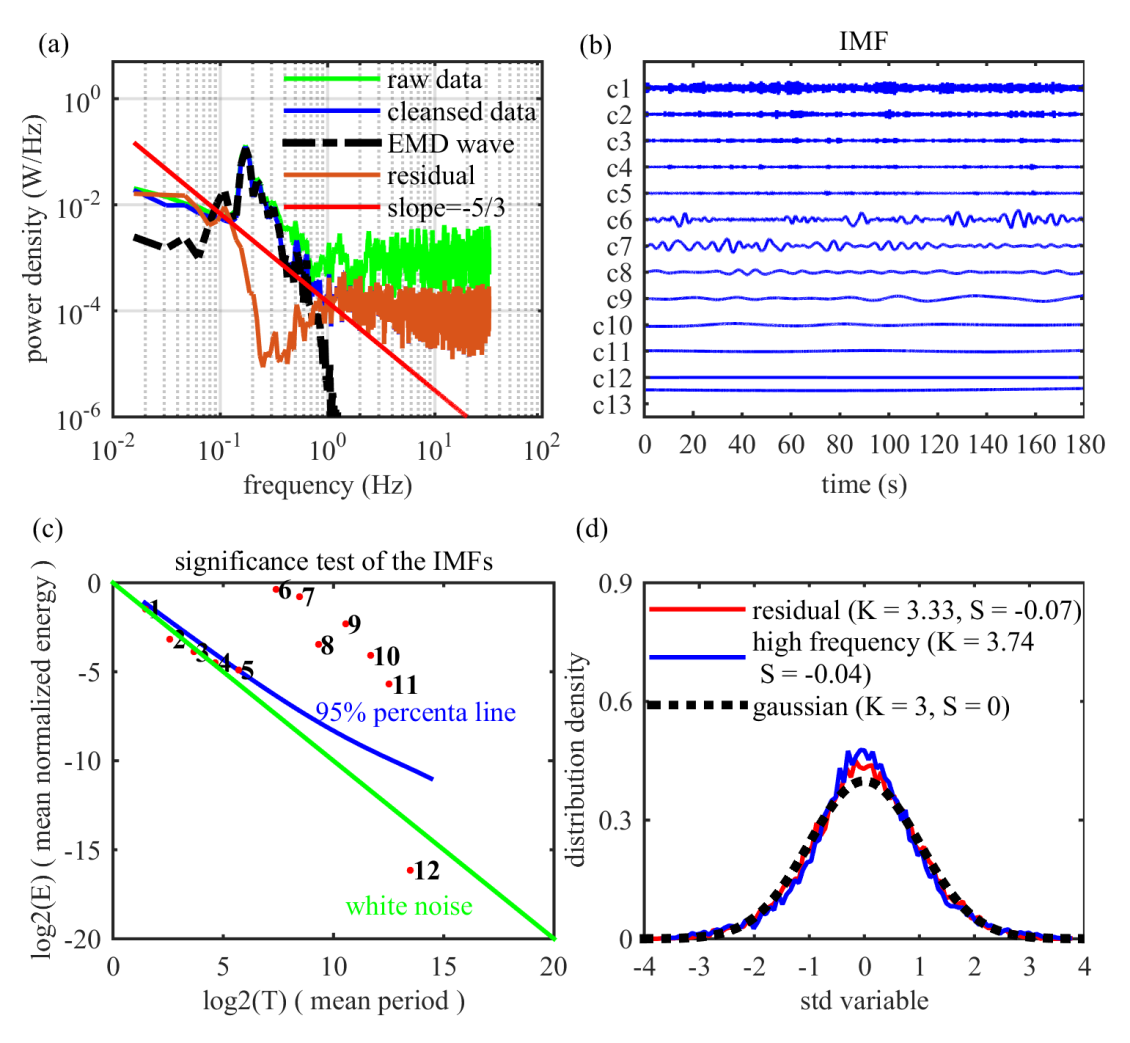


Fig. S13


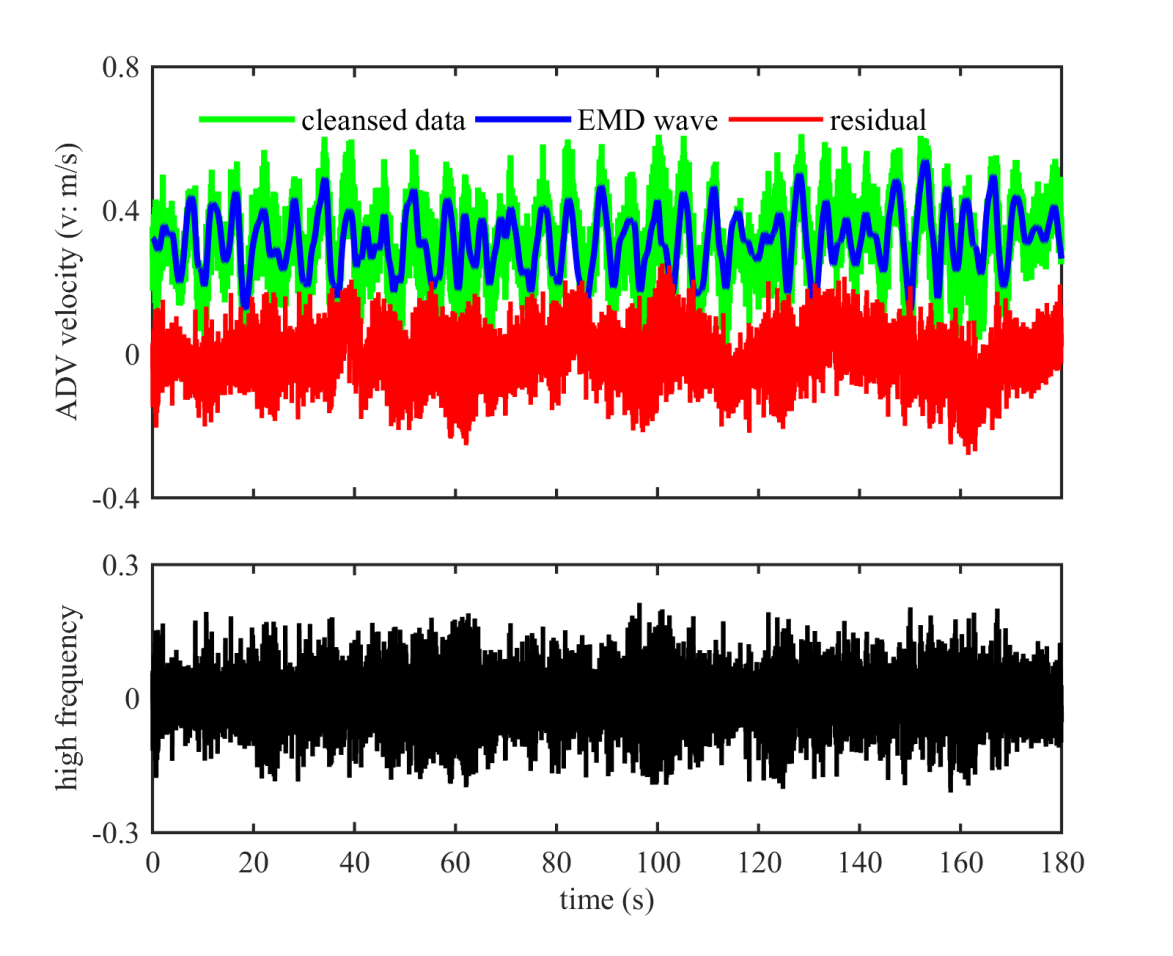


Fig. S14


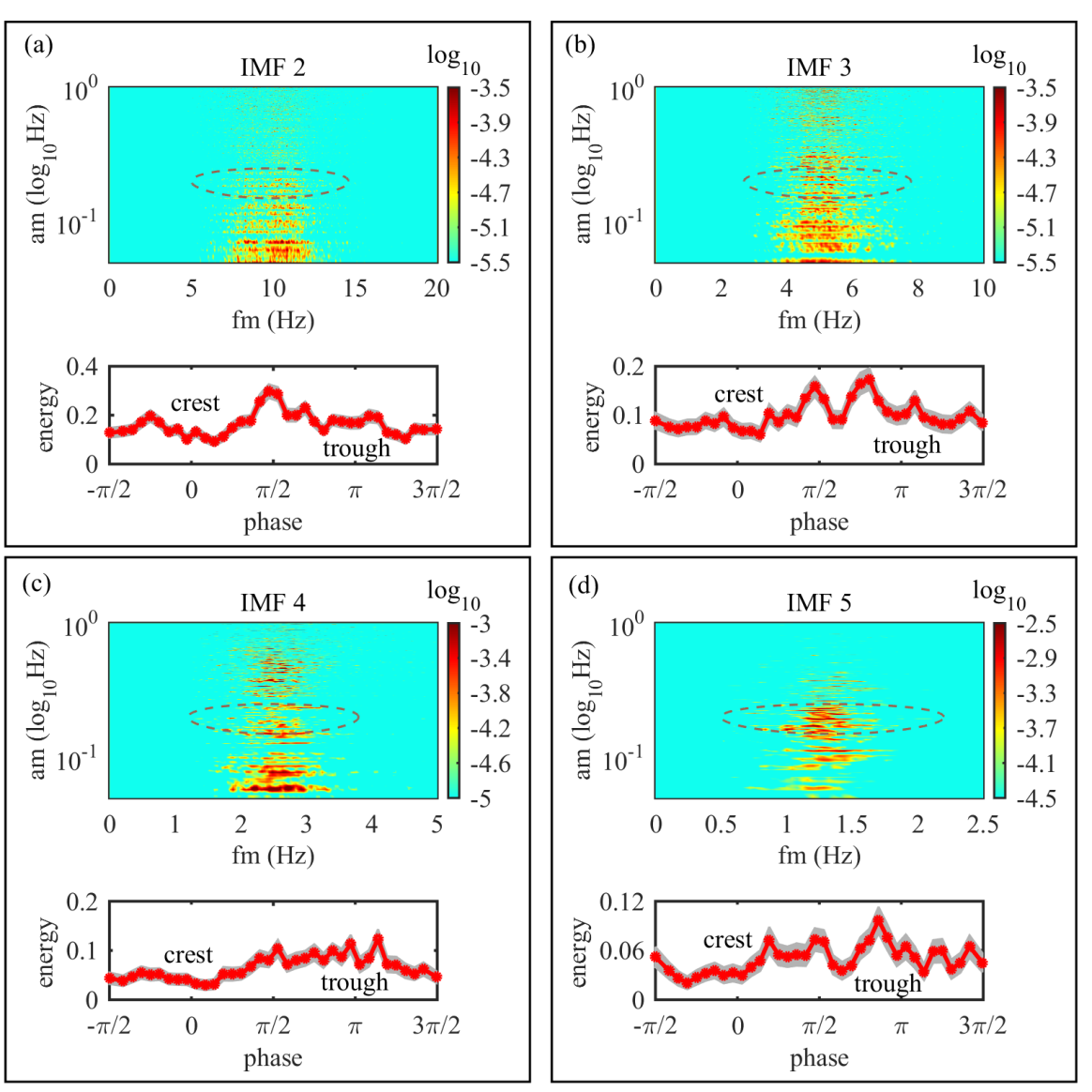


Fig. S15


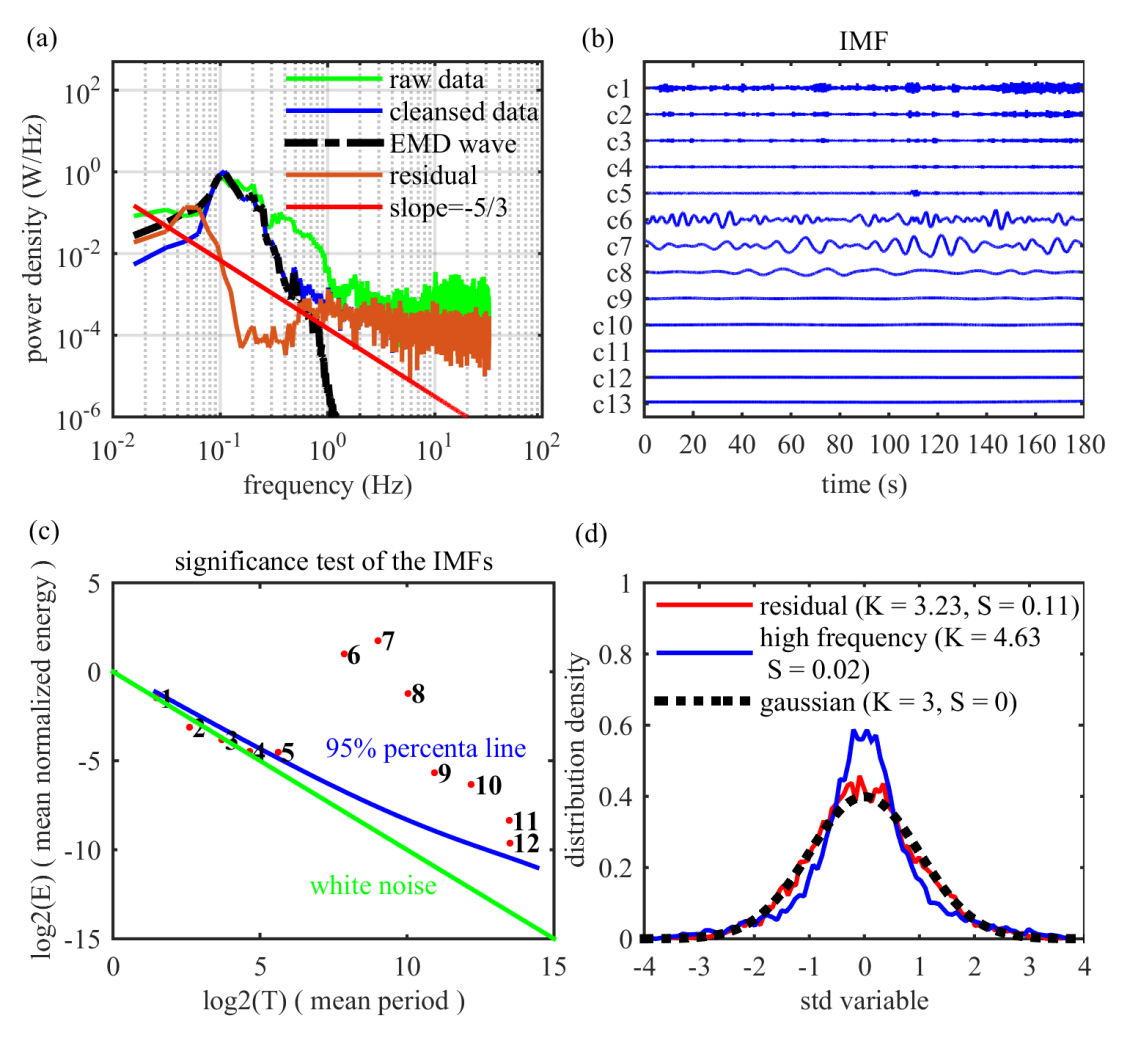


Fig. S16


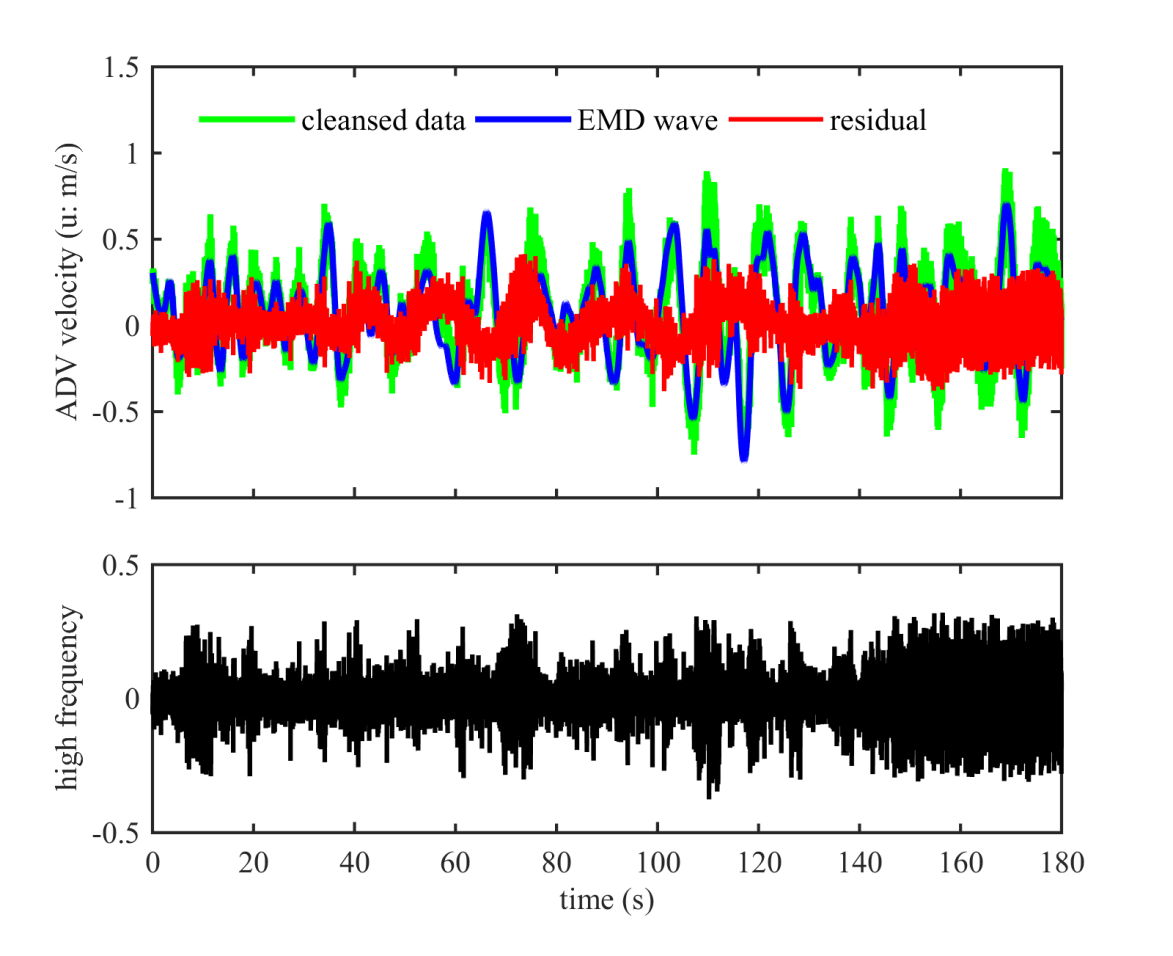


Fig. S17


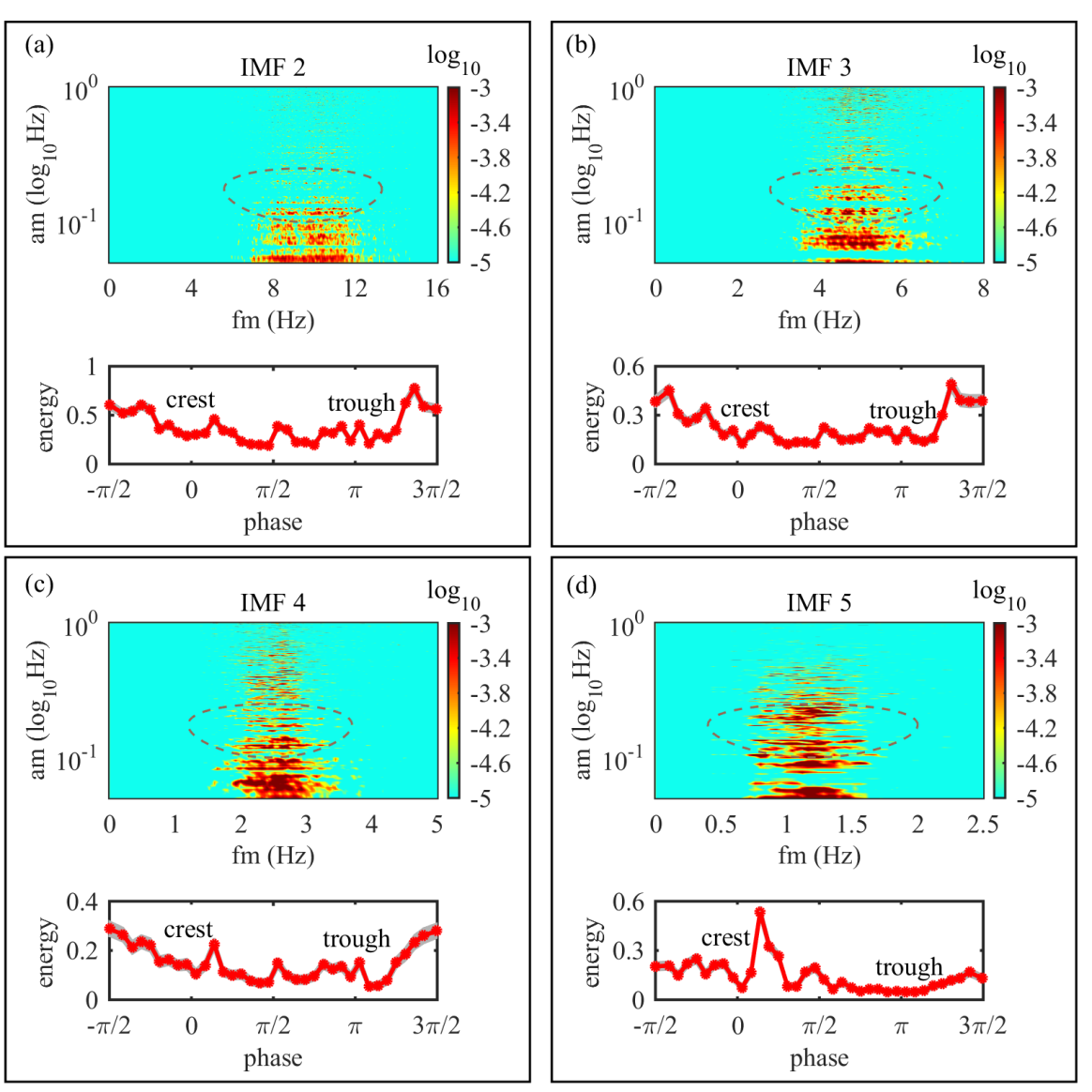


Fig. S18


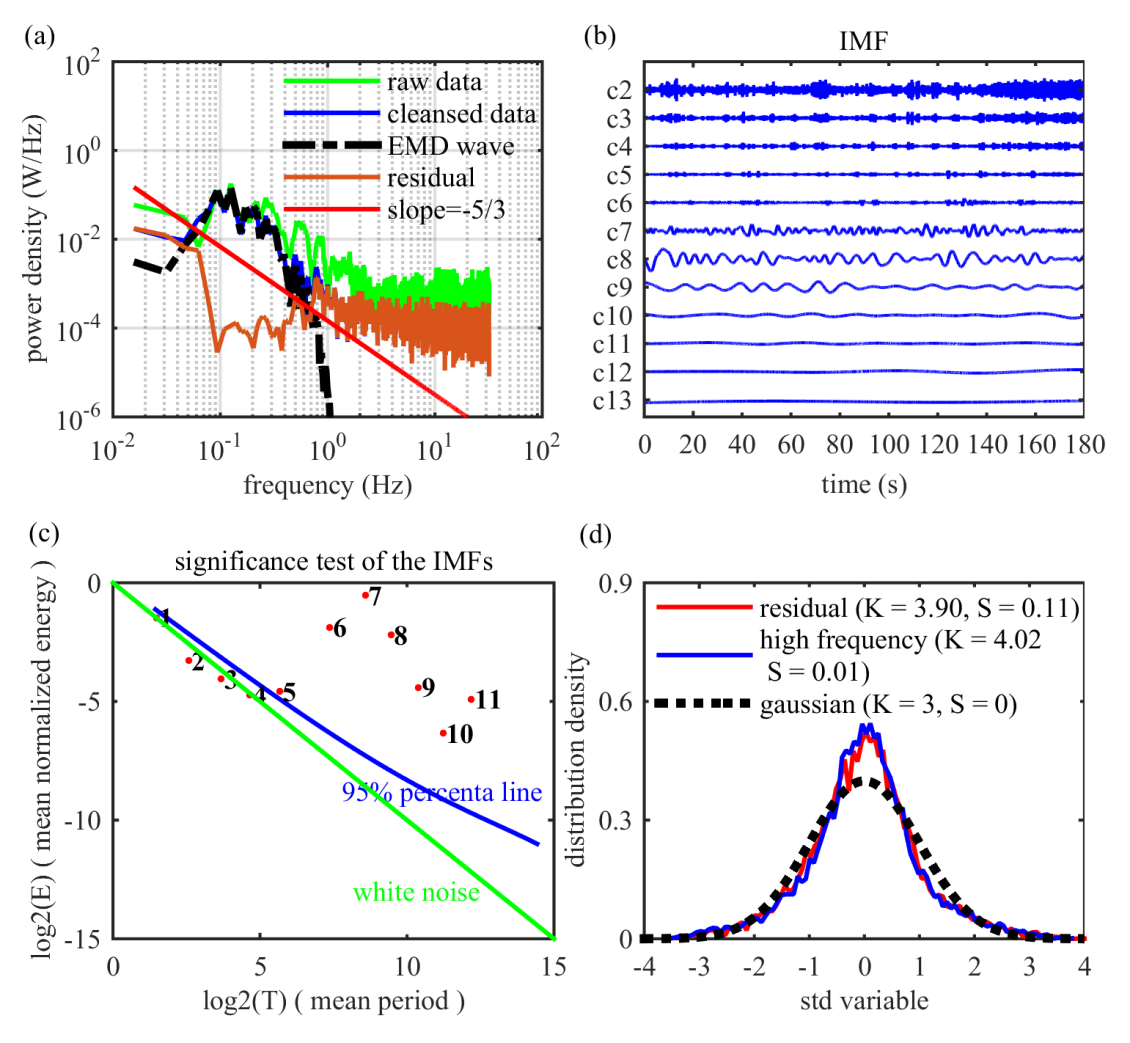


Fig. S19


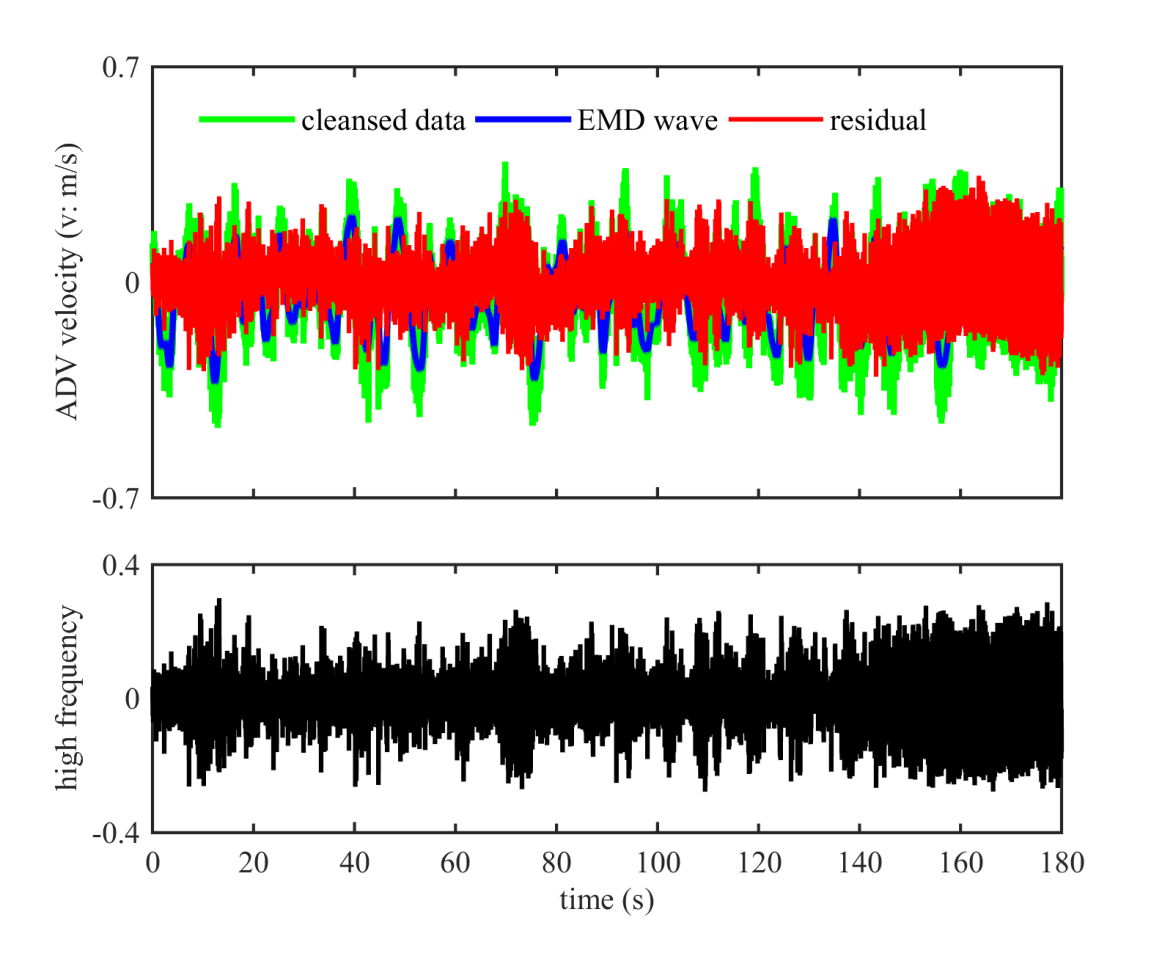


Fig. S20


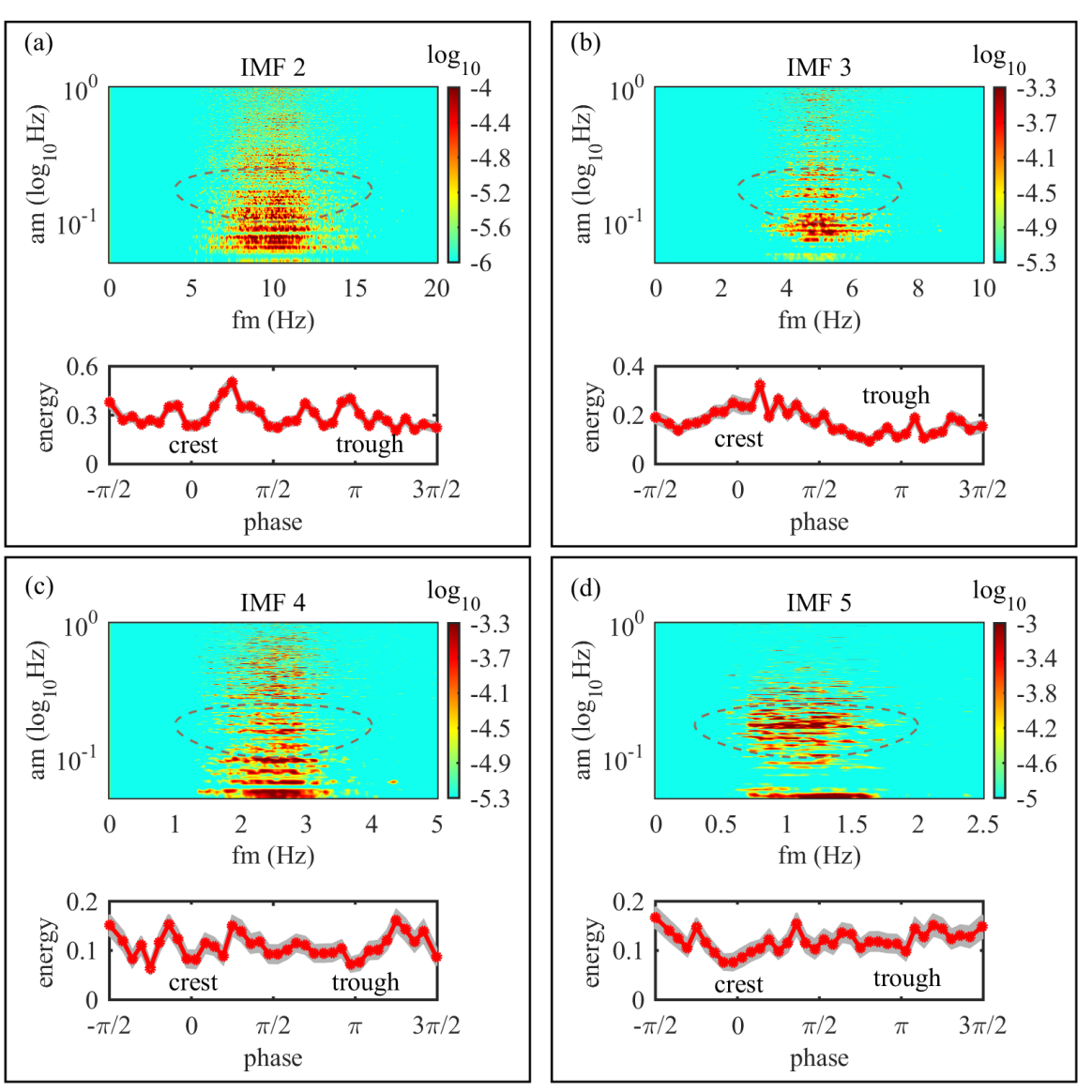


Fig. S21


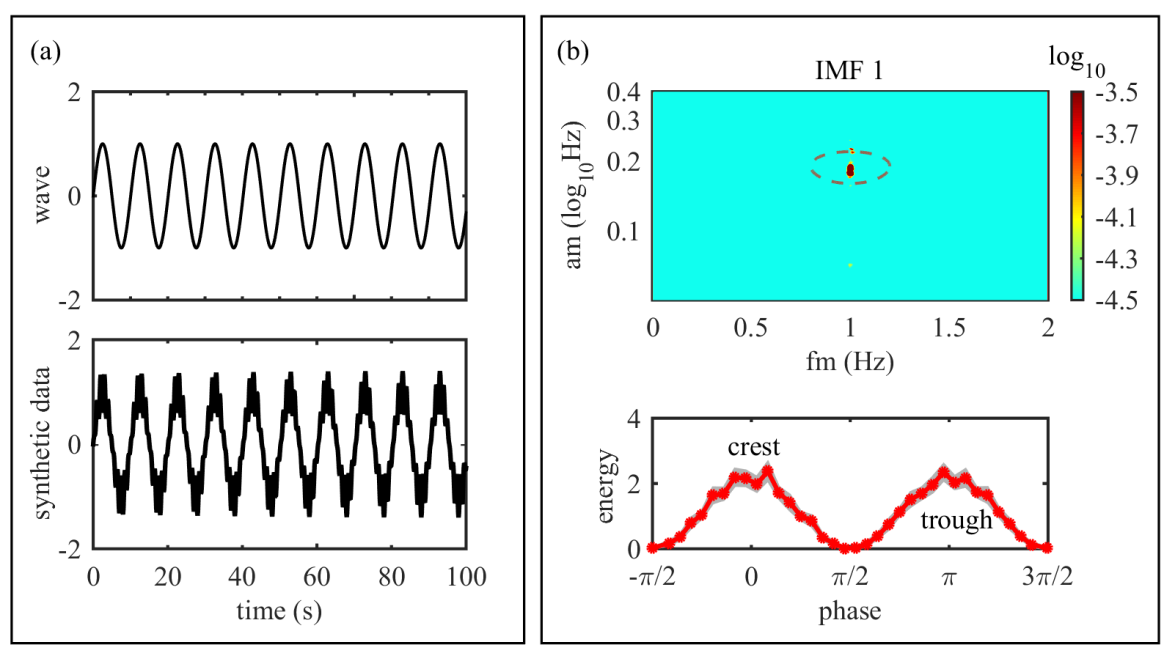


Fig. S22


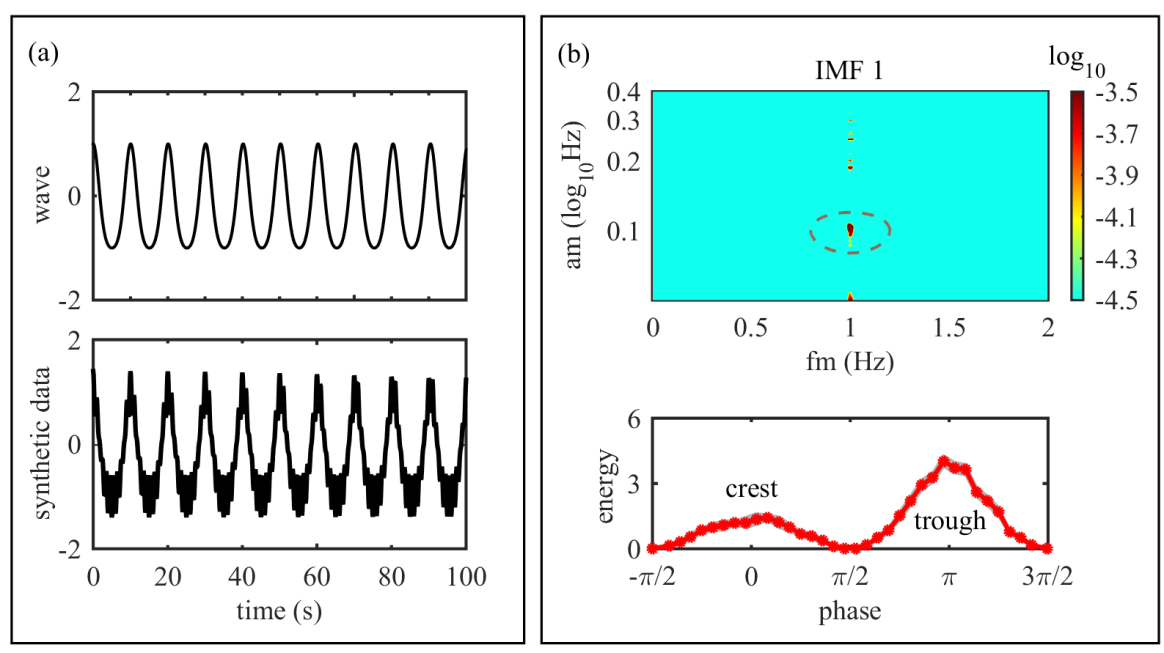


Fig. S23


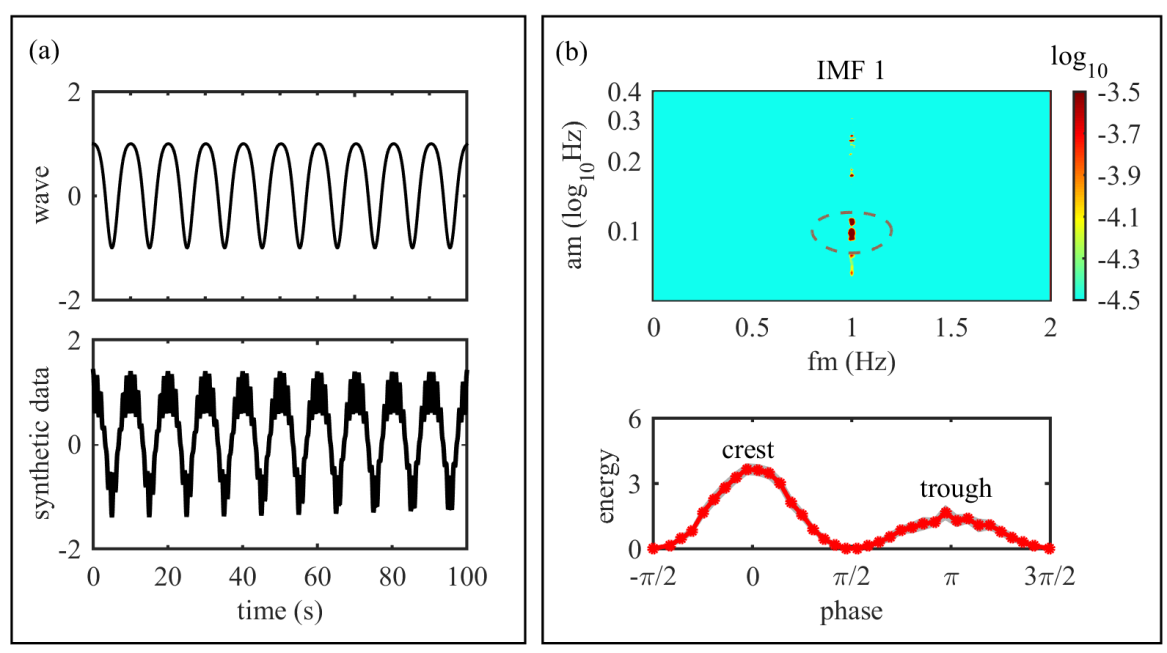


Fig. S24
